# Mammillothalamic Disconnection Alters Hippocampocortical Oscillatory Activity and Microstructure: Implications for Diencephalic Amnesia

**DOI:** 10.1523/JNEUROSCI.0827-19.2019

**Published:** 2019-08-21

**Authors:** Christopher M. Dillingham, Michal M. Milczarek, James C. Perry, Bethany E. Frost, Greg D. Parker, Yaniv Assaf, Frank Sengpiel, Shane M. O'Mara, Seralynne D. Vann

**Affiliations:** ^1^School of Psychology, Cardiff University, Cardiff CF10 3AT, United Kingdom,; ^2^Trinity College Institute of Neuroscience, Trinity College Dublin, Dublin 2, Ireland,; ^3^EMRIC, Cardiff University, Cardiff CF10 3AX, United Kingdom,; ^4^George S. Wise Faculty of Life Sciences, Tel Aviv University, Tel Aviv, Israel 6997801, and; ^5^School of Biosciences, Cardiff University, Cardiff CF10 3AX, United Kingdom

**Keywords:** mammillary bodies, MRI, paradoxical sleep, phase-amplitude coupling, rat, theta

## Abstract

Diencephalic amnesia can be as debilitating as the more commonly known temporal lobe amnesia, yet the precise contribution of diencephalic structures to memory processes remains elusive. Across four cohorts of male rats, we used discrete lesions of the mammillothalamic tract to model aspects of diencephalic amnesia and assessed the impact of these lesions on multiple measures of activity and plasticity within the hippocampus and retrosplenial cortex. Lesions of the mammillothalamic tract had widespread indirect effects on hippocampocortical oscillatory activity within both theta and gamma bands. Both within-region oscillatory activity and cross-regional synchrony were altered. The network changes were state-dependent, displaying different profiles during locomotion and paradoxical sleep. Consistent with the associations between oscillatory activity and plasticity, complementary analyses using several convergent approaches revealed microstructural changes, which appeared to reflect a suppression of learning-induced plasticity in lesioned animals. Together, these combined findings suggest a mechanism by which damage to the medial diencephalon can impact upon learning and memory processes, highlighting an important role for the mammillary bodies in the coordination of hippocampocortical activity.

**SIGNIFICANCE STATEMENT** Information flow within the Papez circuit is critical to memory. Damage to ascending mammillothalamic projections has consistently been linked to amnesia in humans and spatial memory deficits in animal models. Here we report on the changes in hippocampocortical oscillatory dynamics that result from chronic lesions of the mammillothalamic tract and demonstrate, for the first time, that the mammillary bodies, independently of the supramammillary region, contribute to frequency modulation of hippocampocortical theta oscillations. Consistent with the associations between oscillatory activity and plasticity, the lesions also result in a suppression of learning-induced plasticity. Together, these data support new functional models whereby mammillary bodies are important for coordinating hippocampocortical activity rather than simply being a relay of hippocampal information as previously assumed.

## Introduction

The medial diencephalon was arguably the first brain region to be linked to amnesia due to the observed pathology within this area in cases of Korsakoff syndrome (Gudden, 1896; Gamper, 1928). The importance of medial diencephalic structures for memory, in particular the mammillary bodies (MBs) and anterior thalamic nuclei, has been further consolidated over the years from patient studies, nonhuman primate, and rodent work (Jankowski et al., 2013; Dillingham et al., 2015; Vann and Nelson, 2015). However, despite the longstanding association between the medial diencephalon and amnesia, there is still no clear consensus as to why this region is so critical. Given the large number of conditions in which medial diencephalic structures are known to be affected (Kopelman, 1995; e.g., Briess et al., 1998; Van der Werf et al., 2000; Kumar et al., 2008, 2009; Denby et al., 2009; Vann et al., 2009; Özyurt et al., 2014; Dzieciol et al., 2017; Savastano et al., 2018; Perry et al., 2019), this lack of knowledge presents a serious shortcoming. According to mnemonic models based on the Papez circuit, the MBs and anterior thalamic nuclei relay information from the hippocampal formation to the cingulate cortex (Papez, 1937; Delay and Brion, 1969). These models place the medial mammillary nuclei and anterior thalamic nuclei downstream from the hippocampus and, as such, attribute little intrinsic importance to these structures; instead, they are merely considered passive relays of hippocampal information (Wolff and Vann, 2019).

An alternative model is that these medial diencephalic regions are functionally upstream from the hippocampal formation, thus providing crucial inputs necessary for memory formation (Vann, 2010). While there is preliminary evidence consistent with this revised model (Vann and Albasser, 2009; Vann, 2013), it is unclear what information is provided by this ascending pathway and how it contributes to hippocampal functioning. One proposal is that the medial mammillary nuclei, by way of their inputs from the Gudden tegmental nuclei, contribute to memory formation by setting hippocampal theta frequency (Vann, 2009). This contrasts with earlier suggestions that the MBs simply act as a relay of hippocampal theta (Kocsis and Vertes, 1994; Tsanov et al., 2011; Dillingham et al., 2015). Alterations in hippocampal theta would be expected to co-occur with changes in cortical and hippocampal theta-gamma coupling (Canolty and Knight, 2010). Given the longstanding association between hippocampal oscillations and learning and memory-related plasticity (Huerta and Lisman, 1993; Orr et al., 2001; e.g., Bikbaev and Manahan-Vaughan, 2008; Tsanov and Manahan-Vaughan, 2009; Nokia et al., 2012), an implication is that MB disconnection would, in addition to altering oscillatory mechanisms, disrupt microstructural plasticity.

To test these predictions, we used a multifaceted approach to assess the impact of MB disconnection on distal areas of the Papez circuit: the hippocampus and retrosplenial cortex (RSC). Mammillothalamic tract (MTT) lesions were used to model diencephalic amnesia, as damage to this tract is the most consistent feature in thalamic infarct patients with accompanying memory impairments (Clarke et al., 1994; Van der Werf et al., 2003; Yoneoka et al., 2004; Carlesimo et al., 2007, 2011). Furthermore, this fiber tract only innervates the anterior thalamic nuclei, thus removing the possibility that any of the distal effects under investigation are simply driven by direct deafferentation of the hippocampus and RSC. We assessed oscillatory activity both within and across the hippocampus and RSC during theta-rich states, including locomotion and paradoxical sleep (PS) (Leung et al., 1982). Hippocampal and retrosplenial dendritic spine density and clustering and the number and complexity of doublecortin (DCX)-positive neurons were quantified to provide a measure of microstructural plasticity. To assess the timescale of microstructural changes, we performed a complementary multitime point MRI study looking at the effects of MTT lesions on gray matter diffusivity. Together, these convergent approaches enable us to test current models of medial diencephalic function to determine whether they support hippocampal processes that may underpin their contribution to mnemonic processing.

## Materials and Methods

### 

#### Animal subjects

A total of 101 male rats from four cohorts were used across three experiments: 75 Lister-Hooded (Cohorts 1 and 3, Envigo; Cohort 2a, Harlan) and 26 Dark Agouti (Cohort 2b, Harlan). Group comparisons were always made within cohorts (i.e., no cross-strain or cross-supplier comparisons were made). Decisions relating to strain and/or supplier were made based on availability with a focus on reducing animal usage where possible. Previous work from our laboratory has not identified any systematic strain or supplier differences in behavior or in distal effects following lesions to the structures under investigation.

##### Experiment 1: oscillatory activity (Cohort 1).

Twenty-five naive male Lister-Hooded rats (Envigo) were used weighing 300–350 g at the time of surgery. Of these animals, 8 were completely excluded from this study due to misplaced electrodes (*n* = 3), incomplete MTT lesions (*n* = 1), or technical issues resulting from attempts to cohouse after surgery (*n* = 4). Of the remaining 17 rats, 8 received complete bilateral MTT lesions, electrode implantation in CA1 and RSC, as well as chronic placement of EMG electrodes. Five surgical controls received the same electrode configuration without MTT lesions, an additional 4 received electrode implantation in CA1 (*n* = 2) or RSC (*n* = 2) but were also implanted in the claustrum for participation in a separate study.

##### Experiment 2: hippocampal spine and DCX analyses (Cohorts 2a and b).

Naive male Lister-Hooded rats were used for the Golgi spine analyses (Cohort 2a; Harlan) weighing 230–300 g at the time of surgery. Fourteen rats received bilateral MTT lesions and 12 underwent control surgery. Five animals had sparing of the MTT and were removed from all subsequent analyses. For the DCX analyses (Cohort 2b), Dark Agouti rats (Harlan) were used weighing 190–225 g at the time of surgery. Sixteen animals received bilateral MTT lesions and 8 underwent control surgery. Four of the 16 rats had bilateral sparing and were removed from subsequent analyses. One lesion case was excluded from the DCX cell number estimates analysis due to large air bubbles obscuring part of the dentate gyrus on a number of sections.

##### Experiment 3: MR diffusivity (Cohort 3).

Twenty-six naive male Lister-Hooded rats (Envigo) were used; they were ~10 weeks old at the start of the experiment (time of first scan) and weighed 230–280 g at the time of surgery. Sixteen animals received bilateral MTT lesions, and 10 underwent control surgery. Three animals had sparing of the MTT and were removed from all subsequent analyses; 2 control animals died during the study and MR data from 1 control animal could not be recovered. The study spanned 6 months such that at the time of the final scan the animals were ~8 months old.

Animal husbandry and experimental procedures for Experiment 1 were performed in accordance with the European Community directive, 86/609/EC, and the Cruelty to Animals Act, 1876, and were approved by the Comparative Medicine/Bioresources Ethics Committee, Trinity College Dublin, and followed LAST Ireland and international guidelines of good practice. Experiments 2 and 3 were performed in accordance with the UK Animals (Scientific Procedures) Act, 1986 and associated guidelines, the EU directive 2010/63/EU, as well as the Cardiff University Biological Standards Committee.

#### Surgical procedures

All surgeries were performed under an isoflurane-oxygen mixture (induction 5%, maintenance 2%–2.5% isoflurane) during the light phase of a 12 h, day/night cycle. Once anesthetized, animals were positioned in a stereotaxic frame (David Kopf Instruments).

##### MTT lesions.

With bregma and lambda level, the scalp was incised to expose the skull. Bilateral craniotomies were then made at positions targeting the MTT (AP: bregma −2.5 mm; ML: bregma ±0.9 mm). A thermocouple radiofrequency electrode (0.7 mm tip length, 0.25 mm diameter; Diros Technology) was lowered into position (−6.9 mm from top of cortex), and radiofrequency lesions were made (70°C/33 s using an Owl Universal RF System URF-3AP lesion maker; Diros Technology). For control surgeries, the probe was lowered to 1.0 mm above the target to avoid damaging the tract; no lesion was made.

##### Electrode implantation.

If the surgery involved electrode implantation, the skull was cleaned before 5–6 screws were secured and cemented to the skull. Craniotomies were made before careful removal of the dura and subsequent implantation of electrodes in positions corresponding to the following coordinates (mm from bregma unless stated): (CA1: AP: −3.6, ML: ±3.4, DV: −1.9 from top of cortex with a 18.5° angle toward the midline; RSC: AP: −3.6, ML: ±1.1, DV: −1.2 from top of cortex with a 24° angle toward the midline). Rats were then implanted with combinations of 6 or 7 tetrode bundles (25 μm platinum-iridium wires; California FineWire) mounted onto a drivable 32-channel microdrive (Axona). An additional 1–2× custom-made twisted bipolar electrodes (70 μm stainless steel) were incorporated into the remaining microdrive channels via 34 AWG, PTFE-insulated wire (Omnetics). The same wire was adapted for EMG recordings from beneath the nuchal muscle. Implanted microdrives and bipolar electrodes were secured in place using dental cement (Simplex Rapid; Dental Medical Ireland).

The scalp was then sutured, and antibiotic powder was topically applied (Acramide: Dales Pharmaceuticals). Animals were rehydrated with a subcutaneous 5–10 ml injection of 4% glucose saline and given postoperative analgesia (0.06 ml, 5 mg/ml meloxicam, Boehringer Ingelheim).

Following surgery, implanted rats were housed singly while all other rats were housed in small groups of 2–4 rats per cage under diurnal light conditions (14 h light/10 h dark) with free access to water and environmental enrichment. During behavioral testing, animals were food-deprived to within 85% of their free feeding weight.

#### Behavioral experiments

##### T-maze (Cohorts 2a and b).

Before testing, all animals in Experiment 2 were given 2 d of habituation to the T-maze. Animals were then given either 10 (Cohort 2a) or 6 (Cohort 2b) sessions of training. One session was conducted each day, with eight trials in each session. Each test trial consisted of a forced “sample” phase followed by a “choice” phase. During the forced sample phase, one of the goal arms of the T-maze was blocked. After the rat turned into the preselected goal arm, it received a reward. Animals were then returned to the start arm for 10 s. During the choice phase, animals were given free choice to enter either arm, only receiving a reward if the direction opposite to the forced choice in the sample run was chosen (i.e., nonmatching to sample choice). Left/right allocations for the sample runs were pseudo-randomized over daily trials, sessions, and rats, with no more than three consecutive sample runs to the same side in each session. The start arm was kept constant for the whole procedure.

##### Radial-arm maze (Cohort 3):

Animals first received four habitation sessions, which involved unrestricted exploration of all arms of the maze, each containing a scattering of sucrose pellets (45 mg; Noyes Purified Rodent Diet). Rats were trained on a working memory version of the radial-arm task that involved the retrieval of sucrose pellets from each of the arms of the maze. At the start of the trial, all arms were baited, and the animal was placed on the center platform and allowed to make an arm choice. After returning to the center platform, all doors were shut for 10 s before being reopened, and the animal could then make another arm choice. This procedure continued until all arms had been visited or 10 min has elapsed. Rats received two trials/session every other day, totaling 32 trials over 16 sessions.

#### Perfusion/histology

At the end of behavioral experiments, animals were given an overdose of sodium pentobarbital (60 mg/kg, Euthatal, Rhone Merieux) and transcardially perfused with 0.1 m PBS followed by 4% PFA in 0.1 m PBS. Brains from all cohorts other than for Cohort 2a were removed and postfixed in 4% PFA for 4 h before being transferred to 25% sucrose in distilled water overnight. On the following day, 40 μm sections were cut in the coronal plane using a freezing-stage microtome. One 1-in-4 series was collected directly onto gelatin-coated slides and Nissl-stained (cresyl violet, Sigma-Aldrich) for verification of lesion location. The remaining series were reacted for DCX (rabbit polyclonal, Abcam; 1:1000 dilution; Cohort 2b only) and calbindin (D28k; mouse monoclonal; 1:10,000; Swant).

##### Golgi staining (Cohort 2a).

Golgi staining of the whole brain was performed according to the manufacturer's instructions (FD Rapid GolgiStain Kit; FD NeuroTechnologies). Mounted 150 μm coronal sections cut on a cryostat were counterstained with cresyl violet, dehydrated in an ascending series of alcohols, cleared in xylene, and coverslipped using DPX mounting medium (Thermo Fisher Scientific). Slides were recoded by a researcher not involved in data collection to allow blinded analyses.

##### Immunohistochemistry.

Sections were washed 4 × 10 min in 0.1 m PBS containing 0.2% Triton X-100 (PBST) between each incubation period. Endogenous peroxidase activity was quenched in a 0.3% hydrogen peroxide solution (0.3% H_2_O_2_, 10% methanol, and distilled water) for 5 min. Sections were then incubated in 0.1 m PBS containing 3% normal serum for 1 h followed by a 48 h primary antibody incubation at 4°C with 1% normal serum. Following 3 × 10 min PBST washes, sections were reacted for 2 h in a 1:250 secondary solution containing 1% normal serum. After a further 3 × 10 min PBST washes, sections were reacted for 1 h in an avidin/biotin-peroxidase complex solution with 1% normal serum (ABC Elite, Vector Laboratories). Sections were then washed twice in 0.05 m Tris buffer, and the label was visualized with DAB Substrate Kit (Vector Laboratories) according to the supplier's protocol. Sections were then mounted on gelatin-coated slides, dehydrated in an ascending series of alcohols, cleared in xylene, and coverslipped with DPX mounting medium. Slides were recoded by a researcher not involved in data collection to allow blinded analyses.

#### Experiment 1: electrophysiology

Recordings were performed during the light phase of a 12 h, day/night cycle. Following a recovery period of no less than 8–10 d, rats (Cohort 1) were trained to retrieve sugar pellets from wells in four end compartments of a bowtie maze to promote locomotion over a broad range of speeds. Once rewards were retrieved from one end of the maze, a manually operated middle door was opened allowing the animal to investigate and retrieve rewards from the opposite end. Local field potential (LFP) recordings were obtained using the DacqUSB acquisition system Axona Ltd., St. Albans, UK at a sampling frequency of 4.8 kHz and downsampled to 960 Hz and standardized within each recording session (subtraction of the mean and division by the SD of the dataset) for all further analyses. Animal positional information was obtained using a light bar mounted with infra-red LEDs, one larger than the other, and sampled at 250 Hz using a ceiling mounted infrared video camera. Positional information was interpolated to match the sampling frequency of the downsampled LFP. Both before and after recordings in the bowtie maze, rats were socialized with littermates for an average of 1–2 h to provide natural sleep deprivation, following which recordings were made while rats were in a familiar square home arena where they could sleep.

All analyses were performed using custom scripts in MATLAB (version 2018b; The MathWorks).

##### Power spectral density (PSD)/coherence.

Welch's method (*pwelch* function) was used for calculation of frequency domain spectral power, and the coherence between signals was estimated using magnitude-squared coherence (*mscohere* function). Power spectra were normalized by dividing each datapoint by the sum of the power in the spectrum.

##### Running speed analysis.

Running speed was calculated based on the change in position over time calculated from the animal's positional information (sampled at 200 Hz). Running speed was first resized through cubic interpolation to match the sampling frequency of the LFP (960 Hz). The average running speed within 600 ms windows of LFP was calculated for individual recording sessions. Windows were then sorted and assigned speed values based on nearest-integer rounding. Window bins of common speed were concatenated, and speed bins of <2 s combined-duration were discarded. Five, 1.8 s windows of each speed bin were randomly extracted for subsequent analyses.

##### Theta cycle asymmetry.

Determination of the phase of a signal using bandpass filtering and the Hilbert transformation, for instance, temporally homogenizes the oscillatory activity, masking cycle asymmetry (Buzsáki et al., 1985; Belluscio et al., 2012). To preserve and measure oscillatory asymmetry, theta cycle windows were identified first through identification of zero crossing time points in LFP signal that had been bandpass filtered between 4 and 12 Hz. Zero crossing time points were then used to extract start and endpoints for positive (0–180°) and negative oscillation phases (180–360°), which were in turn used to extract windows of the same LFP to which a broader bandpass filter had been applied (0.5–80 Hz). Within positive and negative amplitude windows, the time points of maximum and minimum amplitudes, respectively, were extracted and rescaled to give peak and trough times from which ascending (trough-to-peak) and descending (peak-to-trough) durations were calculated. The absolute difference in ascending and descending durations provided an asymmetry index.

##### PS detection.

PS is characterized by high theta power, low delta power, as well as muscle atonia. Episodes of PS were indexed by initially identifying and excluding awake periods in which rats were moving (running speed >0). A moving window (12 s with 50% overlap) based multitaper method (Mitra and Bokil, 2008) was used on bandpass-filtered (1–60 Hz) LFP for time-domain calculation of spectral power. A threshold based on the theta (4–12 Hz)-delta (1–4 Hz) power ratio (TD ratio) was then calculated and epochs of slow wave sleep and PS were characterized by a low TD ratio (high delta, low theta power), and high TD ratio, respectively. Episodes of putative PS (high TD ratio; minimum 15 s in duration) were validated using a threshold set for the root mean square of the synchronously recorded EMG signal.

##### Phase-amplitude coupling.

Phase-amplitude coupling was calculated using the approach of Tort et al. (2010). Briefly, theta phase (2° bins ranging from 0 to 20°) was calculated through the Hilbert transformation, creating a homogenized theta cycle from which gamma/high-frequency oscillations (HFOs) ranging from 30 to 200 Hz (in 5 Hz bins), were compared. An adaptation of the Kullback-Leibler distance was used to generate a modulation index (MI), derived from a phase-amplitude plot.

#### Experiment 2: Golgi and DCX analysis

##### Golgi Sholl analysis (Cohort 2a).

Image stacks were obtained with an LSM 510 confocal microscope (Carl Zeiss) using a 20× apochromat objective. A Helium-Neon laser (633 nm) was used to image the slices at high resolution (1024 × 1024 pixels). Images were acquired at preset intervals (0.5 μm) on the *Z* plane, so generating image stacks (~120–140 images per stack) that allowed the analysis of dendritic arbors in three dimensions. ImageJ (1.48a Fiji version, National Institutes of Health) (Schindelin et al., 2012) and its free segmentation plugin Simple Neurite Tracer (Longair et al., 2011) were used for semiautomated tracing and Sholl analysis of dendritic arbors. The analysis involved overlaying the cell with a series of concentric spheres spaced 1 μm apart, centered on the cell body, and counting the number of dendritic arbors intersecting each circle relative to the distance from the soma to a maximum distance of 300 μm for RSC neurons and 350 μm for CA1 and DG neurons.

Eligible arbors were required to be intact, clearly visible, clear of artifacts, and well isolated from neighboring neurons. In RSC, Sholl analyses were performed on the dendritic arbors of small fusiform and canonical pyramidal neurons whose somata were positioned in superficial layers II-III of the rostral portion of RSC, whereas in CA1, basal arbors of CA1 pyramidal neurons were used. In the DG Sholl, analyses were performed on neurons whose somata were located in the granule cell layer and whose dendritic arbors were projecting toward the molecular layer.

Dendritic arbors were traced from a total of 476 cells: 262 from surgical control brains and 214 from MTT-lesioned brains with at least five arbors for each of apical RSC, basal RSC, basal CA1, and DG per animal. For RSC arbors, identifying cells with both apical and basal segments that were eligible for tracing was rare (7 from the MTT lesion group and 1 from the control group) due to staining artifacts or overlapping of soma or dendrites from neighboring neurons. Specifically, 73 basal RSC arbors and 77 apical RSC arbors for the Sham group and 46 basal RSC arbors and 54 apical RSC arbors for the MTT lesion group were traced. For hippocampal arbors, 59 CA1 and 53 DG arbors for the Sham group and 65 CA1 and 49 DG arbors for the MTT lesion group were traced. The mean number of intersections per animal was then calculated for each ROI.

##### Dendritic spine counts (Cohort 2a).

**Figure 1.**
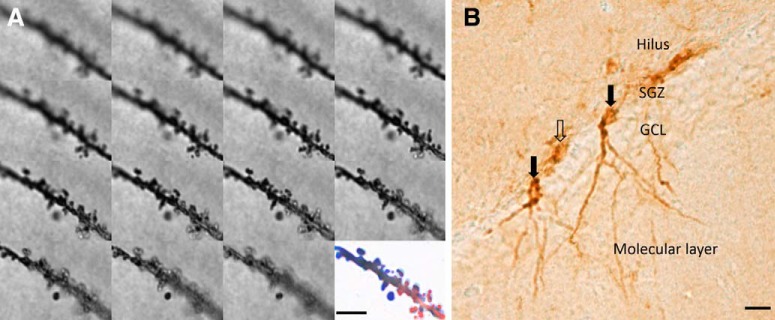
Representative examples of an image stack from Golgi-stained dendritic segment of a CA1 basal arbor before and after image processing used for subsequent spine density and clustering analysis (***A***) and DCX-positive cell types (***B***). Unfilled arrow points to an immature DCX cell. Filled arrows indicate mature DCX cells used in Sholl analysis. GCL, Granule cell layer; SGZ, subgranular zone. Scale bars: ***A***, 5 μm; ***B***, 20 μm.

Image stacks were acquired with a DM6000 microscope with a digital camera (Leica Microsystems, DFC350 FX) and Leica Microsystems Application Suite imaging software. A 100× (NA 1.4) oil-immersion objective using transmitted light was used to collect 30–90 images per stack with a 0.2 μm step size. Dendritic segments of between 20 and 25 μm were traced and cropped with the Simple Neurite Tracer plugin (Longair et al., 2011) according to the eligibility criteria adapted from Harland et al. (2014): one segment per neuron was counted; segments did not belong to the primary branch; segments were unobscured by other dendrites or staining artifacts; segments started and ended equidistant between two spines and started at least 10 μm from any terminal or branching points. Stacks of images were then processed using a custom ImageJ (Fiji version 1.51 h, https://imagej.net/) macro. Briefly, stacks were filtered and sharpened and collapsed into a single 2D projection plane for manual counting of spines (Fig. 1*A*). Cartesian coordinates of identified spines were transformed to map onto a linear representation of the dendritic branch and spine density (number of spines/10 μm dendrite length) and mean nearest neighbor (distance to the nearest spine in one-dimensional space) were calculated. In total, 249 RSC apical and 347 CA1 basal segments were included (4579 RSC spines; 9242 CA1 spines). The mean spine density and nearest neighbor per animal were then calculated for each ROI.

##### DCX Sholl analysis (Cohort 2b).

Only mature (late phase) cells (Mandyam et al., 2008) were analyzed (Fig. 1*B*); the inclusion criteria were as described above. Plane images were captured under bright-field microscopy with a Leica Microsystems DMRB microscope equipped with a 20× (NA 0.50) objective, a CCD camera (Olympus, DP73), and Cell Sense Dimensions software 1.16 (Olympus). Images were converted to 8-bit grayscale and inverted before Sholl analyses (to a maximum distance of 300 μm from the soma). Dendritic arbors were traced from a total of 158 cells: 88 cells for MTT-lesioned cases and 70 cells from surgical controls with 5–16 cells analyzed per case. The mean number of intersections per animal was then calculated.

##### DCX cell number estimates (Cohort 2b).

The unilateral total number of DCX cells was estimated using the optical fractionator method (Gundersen, 1986; West et al., 1991), performed using a Leica Microsystems DM6000 microscope equipped with a motorized stage (BioPrecision, Ludl Electronic Products), *z* axis focus control (Ludl Electronic Products, #99A420), and a CCD camera (CX 9000) connected to a computer running StereoInvestigator 8.0 software (both MicroBrightField). Sampling of sections followed a systematic, uniform random sampling scheme. A section sampling fraction of 1/4 was used, resulting in 15–23 sections sampled per brain (cut section thickness 40 μm; mounted section thickness 13 μm). The contour of the granule cell layer, including the subgranular zone covering the entire dentate gyrus extending 1.80–6.60 mm posterior to bregma (Paxinos and Watson, 1998), was traced live using a 10× objective (0.4 NA), and the cells were counted using a 63× oil-immersion objective (NA 1.4). A dissector height of 8 μm with a 2 μm guard zone was used to sample in the *z* axis. The counting frame area was 2500 μm^2^ (50 × 50 μm), and the *x-y* step length was set to 100 μm (area 10,000 μm^2^). These parameters resulted in a mean of 347 total DCX neurons (range, 266–447) being counted in an average of 639 counting frames (range, 471–817). The number of DCX neurons was estimated from the following:

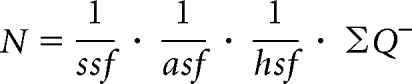
 where *ssf* is the section sampling fraction; *asf*, the area sampling fraction, is the ratio of the counting frame area to the area of the *x-y* step length; *hsf*, the height sampling fraction, is the ratio of the dissector probe height to the *Q*^−^ weighted tissue thickness; and ∑ *Q*^−^ is the total count of cells sampled (Dorph-Petersen et al., 2001).

The sampling method used was sufficiently precise as the calculated coefficient of error (CE) was 0.05 for control and 0.06 for lesion brains (Gundersen et al., 1999). As a measure of the variability of the estimates, the observed interindividual coefficient of variation (CV = SD/mean) was determined as 0.18 for control and 0.14 for lesion brains. The sampling strategy was considered optimal as the biological variation contributed >50% to the observed relative variance, thus providing a reliable indication of the true numbers of DCX cells, that is, $\frac{CE^{2}}{CV^{2}}$ ≤ 0.5 for both the control and lesion group.

#### Experiment 3: MR diffusivity

##### Scans.

Animals in Cohort 3 underwent four MR scanning sessions: 6 weeks before surgery (Scan 1); 9 weeks after surgery (Scan 2); following the second session of radial-arm testing (13 weeks from surgery; Scan 3); and following the final session of radial-arm testing (17 weeks from surgery; Scan 4). Before scanning and behavioral testing, rats were placed in a quiet, dark room for 60 and 30 min, respectively.

Rats were scanned with a 9.4 Tesla MRI machine (Bruker) with a 72 mm, 500 W four-channel transmit coil. The protocol comprised a multigradient structural T2 RARE scan (8 repetitions; 24 slices at 500 μm *z* spacing and an *x-y* resolution of 137 μm; duration: 10 min 40 s), followed by a diffusion tensor imaging (DTI) scan with a diffusion-weighted spin-echo EPI pulse sequence. The DTI acquisition parameters were TR/TE = 4000/23.38 ms, 4 EPI segments, 32 gradient directions with a single b value at 1000 s/mm^2^ and five images with a b value of 0 s/mm^2^ (B0) (Jones et al., 1999). Each scan included 24, 500 μm coronal brain slices with an *x-y* resolution of 273 μm. The EPI acquisition lasted 40 min.

During scan acquisition, rats were lightly anesthetized with 1%–1.5% isoflurane in medical air and placed on a heatmat. The heart rate, breathing rate, and temperature of the animals were monitored throughout each scanning session. The entire scanning protocol lasted just over an hour, and DTI images were acquired within 80–100 min of the endpoint of behavioral testing.

##### Extraction of DTI metrics and normalization procedures.

Diffusion-weighted data were processed through a combination of custom MATLAB (The MathWorks) scripts and the *ExploreDTI* toolbox (Leemans et al., 2009). Briefly, this comprised a motion/distortion correction by coregistration of diffusion-weighted volumes to the initial B0 image (with appropriate b-matrix rotation) (Leemans and Jones, 2009), correction for Gibbs ringing artifacts (Osher and Shen, 2000; Sarra, 2006), and robust tensor fitting (Chang et al., 2005). Subsequently, metrics of fractional anisotropy (FA) and mean diffusivity (MD) (Pierpaoli and Basser, 1996) were extracted.

Image normalization was achieved using the Advanced Normalization Tools software package (Avants et al., 2011). A cohort-specific template was first created through iterative coregistration of FA maps. Coregistration of individual FA volumes to the template was then achieved through symmetric diffeomorphic registration (Avants et al., 2008) with reuse of the resultant warp fields to provide equivalent normalization of MD data. In all cases, a cubic b-spline interpolation scheme was used.

##### Creation of mean hemispheric maps.

Inspection of the generated FA and MD maps revealed asymmetric noise related to the acquisition procedure. To remedy this, mean hemispheric maps were produced (e.g., Kikinis et al., 2019). This approach was deemed appropriate due to the following: (1) the bilateral lesioning of the MTT (Vann and Albasser, 2009); and (2) the assumption that lesion-induced and training-induced changes would not show laterality (e.g., Sagi et al., 2012). The maps were further processed in ImageJ (Fiji version 1.51h, https://imagej.net/). First, the images were resized to a higher resolution (factor of 3) by using bicubic interpolation. Next, coronal slices were flipped horizontally, and pairs of mirror images were iteratively registered to their mean to increase the hemispheric symmetry (rigid registration, followed by affine registration and elastic transformations (Sorzano et al., 2005) (https://imagej.net/BUnwarpJ). The coregistered mirror images were mean-averaged, and a mean FA template was created. The template was then manually masked to restrict the analyses to brain tissue only. All FA and MD maps were finally smoothed using a 0.3 mm 3D Gaussian kernel.

#### Experimental design and statistical analyses

##### Experiment 1.

Statistical analyses were performed in R Statistics (version 3.5.2; provided in the public domain by the R Foundation for Statistical Computing, Vienna, Austria; R Development Core Team, 2009, available at http://www.r-project.org/). General linear models (“lme4” package) (Bates et al., 2015) were fitted for lesion/control group comparisons. For comparisons involving speed/treatment interactions, a random term (1|subject ID) was included to account for repeated measures. Given the difficulties associated with determining degrees of freedom in these instances, *p* values were obtained through likelihood ratio tests comparing nested models (i.e., inclusion of the individual components of the interaction but not the interaction itself). All graphs were generated using the “ggplot2” package (Wickham, 2016), while figures were compiled in Inkscape (Inkscape version 0.92.4, The Inkscape Project, freely available at www.inkscape.org).

##### Experiment 2.

For Sholl analyses, the number of intersections per incrementing radius (binned into 5 μm steps) was counted. For spine counts, spine density was expressed as spines per 10 μm. DCX cell counts are reported as unilateral estimates. For all measures, the mean was derived for each individual animal and this was used for analyses. Parametric tests (ANOVA and *t* test) were used to compare groups. Greenhouse–Geisser adjustments were used to correct for sphericity for repeated-measures analyses, and Welch's *t* test for unequal variances was used where appropriate. One-sample *t* tests comparing overall mean percentages against 50% were used to evaluate whether the lesion and control groups were performing above chance on the T-maze task. SPSS software (version 20, IBM) was used to perform statistical analyses.

##### Experiment 3.

Voxelwise analyses were performed in MATLAB using an ANOVA script based on CoAxLab (2019). A 2 × 4 factorial ANOVA was implemented for FA and MD maps with a between-factor of surgery (control /lesion) and the within-factor of scan (Scan 1 to Scan 4). Since false discovery rate correction eliminated virtually all voxels, except for those in the lesion area, data presented in Figure 8 are uncorrected results and restrict regional analyses to clusters below the α level of 0.005. Such identified voxels in the MTT, hippocampal, retrosplenial, and MB regions were subsequently used to elucidate the timeline of diffusivity changes across the four scans. A 2 × 4 ANOVA was performed for each region (SPSS 25.0, IBM) using raw FA values. In *post hoc* analyses, both the differences from baseline (Scan 1) and between the groups (control/lesion) at each scan time were compared, and Bonferroni-adjusted. For all experiments, unless otherwise stated, the threshold for significance was set at *p* < 0.05.

## Results

### MTT lesions

**Figure 2.**
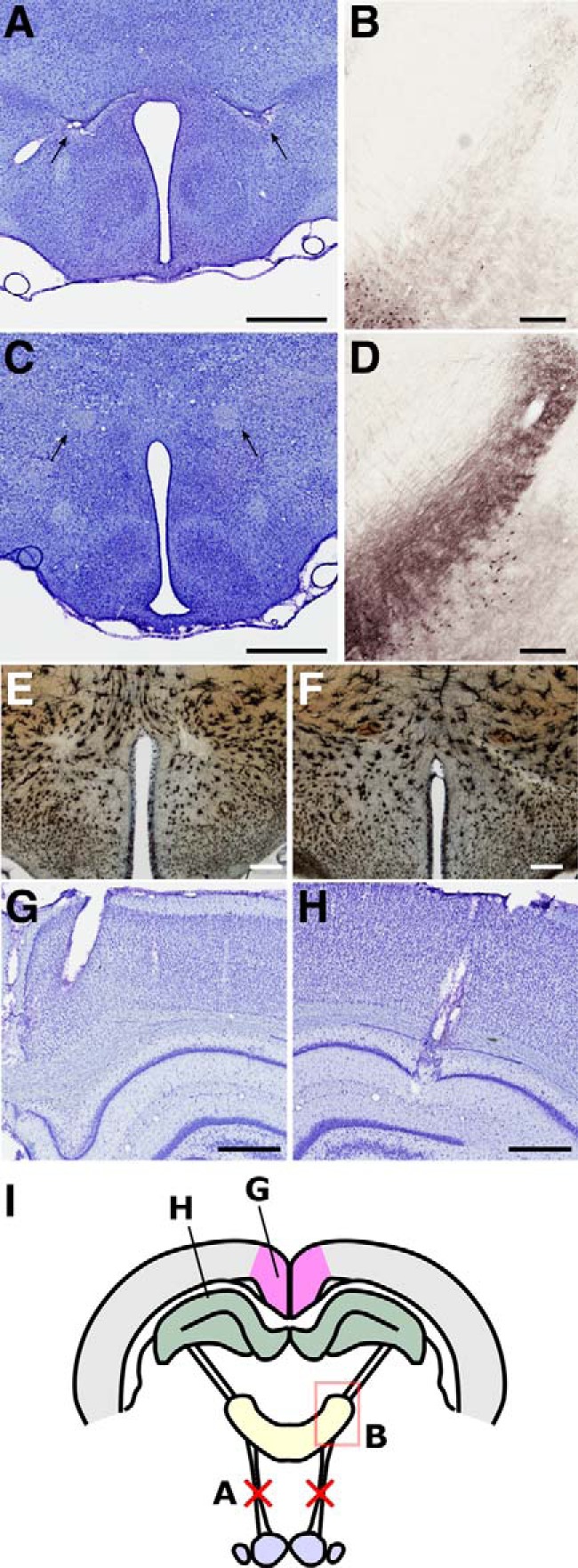
***A***, ***C***, Arrows indicate the location of the MTT in representative lesion and control cases, respectively, whereas lesion-induced reduction in calbindin immunoreactivity in the anteroventral nucleus of the thalamus (***B***) compared with controls (***D***) provided additional verification of lesion success. ***E***, ***F***, Golgi-stained tissue showing the MTT in a control (***E***) and an example MTT lesion (***F***). ***G***, ***H***, Nissl-stained verification of representative electrode placements in the RSC (***G***) and CA1 subfield of the hippocampus (***H***). ***I***, Schematic diagram showing the experimental approach. Red crosses represent the MTT lesions. Scale bars: ***B***, ***D***, 250 μm; all others, 500 μm.

Four separate cohorts of MTT lesion rats were used (for further detail, see Materials and Methods). Following MTT lesions (Fig. 2*A*), calbindin immunoreactivity in the anteroventral thalamic nucleus is reduced relative to controls (Fig. 2*B,D*) which, in addition to Nissl staining (Fig. 2*A,C*), provides further verification of lesion accuracy. While these approaches were not possible in Golgi-stained sections (Cohort 2a), lesion sites in this cohort were verified based on reduced staining density while damage to the MTT was verified by the absence of Golgi deposits in the white matter (Fig. 2*E*,*F*). Only lesions with discrete yet complete bilateral lesions were included in the study. Following lesion verification, the final numbers were as follows: Cohort 1 (Experiment 1: oscillatory activity), 8 lesion, 10 controls; Cohort 2a (Experiment 2a: Golgi), 9 lesion, 12 controls; Cohort 2b (Experiment 2b: DCX), 12 lesion, 8 controls; and Cohort 3 (Experiment 3: MR diffusivity), 13 lesion, 7 controls.

### Experiment 1: oscillatory activity

**Figure 3.**
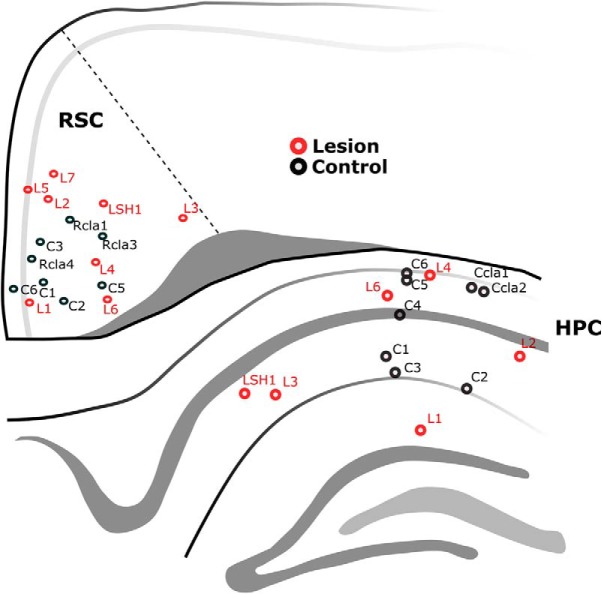
Schematic diagram showing the representative anteroposterior position and anatomical locations of all recording electrodes in the study (30 electrodes in 18 animals). LFP recordings were made from the septal CA1 subregion of the HPC and the septal RSC. Animal identification labels are provided to permit identification of the proportion of the animals that recorded from both regions simultaneously (*n* = 11) and those that were recorded from either region singularly (*n* = 7).

To determine whether mammillothalamic disconnection influenced hippocampal and neocortical oscillatory activity, we recorded LFPs in the RSC (Fig. 2*G*) and the CA1 subfield of the hippocampal formation (HPC; Fig. 2*H*), simultaneously, in 11 rats. Six of these animals had received discrete bilateral lesions of the MTT (Fig. 2*A*, arrows). An additional 7 animals, 2 of which had MTT lesions, were implanted in the RSC alone. The remaining 5 surgical controls were recorded singularly from either brain region (HPC, *n* = 2; RSC, *n* = 3; Fig. 3).

#### MTT lesions attenuate hippocampal theta frequency during locomotion

**Figure 4.**
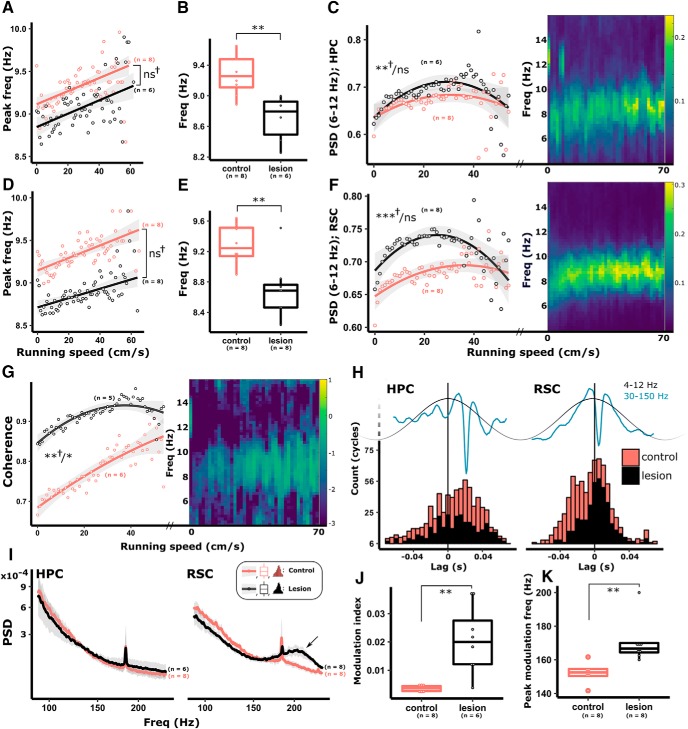
In control animals, both peak theta frequency and theta power (PSD) are modulated by running speed in HPC (***A–C***) and RSC (***D–F***). In animals with MTT lesions, the HPC power-speed relationship was intact (***C***). Although the relationship between peak theta frequency and running speed was intact in lesioned animals (***A***, HPC; ***D***, RSC), peak theta frequency was significantly attenuated across the range of measured speeds in both HPC (***B***) and RSC (***E***). Theta-band coherence between HPC and RSC is positively correlated with running speed (***G***). ***C***, ***F***, ***G***, Heat maps represent higher theta power in HPC, RSC, and theta-band coherence between the two regions, respectively, increasing with speed in individual, representative control animals. Theta-band coherence between HPC and RSC in lesioned animals was significantly greater across speed bins (***G***); however, lesion animals had an intact relationship between theta coherence and running speed (***G***). ***H***, Gamma band (30–150 Hz) trough-triggered averages of theta (4–12 Hz) oscillations were used to calculate the time lag from the theta cycle peak. Consistent with previous studies (Belluscio et al., 2012), gamma power was most strongly associated with the descending phase in HPC and the peak of the theta cycle in RSC with no difference between treatment groups. Gamma power during locomotion was unaffected by MTT lesions; however, increased power in the 180–240 Hz range (HFOs) was observed in RSC (***I***, right, arrow). Corresponding increases in phase amplitude coupling in the corresponding frequency range were observed in RSC (***J***, ***K***), reflecting the increased HFO. **p < 0.01, *p < 0.05. ns, not significant, †Results of speed/treatment interactions. Color key in ***I*** applies to all graphs in the figure. Red represents control. Black represents lesion.

To promote locomotion across an evenly distributed, and wide range of running speeds, animals were trained to retrieve sugar pellets in a bowtie maze (Nelson and Vann, 2017), which resulted in animals consistently reaching speeds of 40–55 cm/s. No significant difference was found between lesion and control groups in the density distributions of speed measurements (0–55 cm/s; *p* = 0.47). Theta (6–12 Hz) power and frequency are both positively correlated with running speed in HPC (Slawinska and Kasicki, 1998; Maurer et al., 2005; Jeewajee et al., 2008; Sheremet et al., 2016; Carpenter et al., 2017). To determine whether disconnection of the MBs from the Papez circuit impaired the encoding of speed in HPC and RSC, we first examined the power and peak frequency of theta oscillations from recordings in awake, locomoting animals. We found that the peak theta frequency (6–12 Hz) of lesioned animals was significantly attenuated across all running speed bins in both the HPC (−0.57 ± 0.15 Hz, *t*_(13)_ = −3.79, *p* = 0.003; Fig. 4*B*) and the RSC (−0.59 ± 0.16 Hz, *t*_(15)_ = −3.63, *p* = 0.003; Fig. 4*E*). As expected, in control animals, the peak theta-band frequency (6–12 Hz) showed highly significant positive linear correlations with running speed in both the HPC (estimate: 0.03 ± 0.00 Hz/(cm/s), *t*_(7)_ = 10.95, *p* < 0.001; Fig. 4*A*) and RSC (estimate: 0.03 ± 0.00 Hz/(cm/s), *t*_(7)_ = 11.42, *p* < 0.001; Fig. 4*D*). MTT lesions attenuated the peak frequency of theta, but they did not disrupt the relationship between running speed and theta frequency; that is, there was no difference between treatment groups in the speed-frequency interaction (lesion group estimate, HPC: −0.00 ± 0.00 Hz/(cm/s), χ^2^ = 0.33, *t*_(13)_ = −0.58, *p* = 0.583 (Fig. 4*A*); RSC: −0.00 ± 0.00 Hz/(cm/s), *t*_(15)_ = −1.71, χ^2^ = 2.92, *p* = 0.087; Fig. 4*D*), reflecting, at least in part, intact glutamatergic/GABAergic septo-hippocampal connections (Fuhrmann et al., 2015; Carpenter et al., 2017).

While running speed and theta frequency exhibited a linear relationship, that of theta band power (6–12 Hz) and running speed was best represented by a quadratic function (Fig. 4*C*,*F*), likely reflecting the plateau in interneuron and pyramidal cell firing rate at higher running speeds (i.e., > 20 cm/s) (Maurer et al., 2005; Ahmed and Mehta, 2012). Theta band power was significantly correlated with running speed in the control animals in both HPC (2.30 × 10^−4^ ± 1.47 × 10^−5^ a.u./(cm/s), *t*_(7)_ = 15.64, χ^2^ = 187.81, *p* < 0.001; Fig. 4*C*) and RSC (2.08 × 10^−4^ ± 1.48 × 10^−5^ a.u./(cm/s), *t*_(7)_ = 14.04, χ^2^ = 157.24, *p* < 0.001; Fig. 4*F*), and no overall difference between theta-band power was found in either region between treatment groups. In both HPC and RSC, the speed/power relationship of lesioned animals was significantly altered in magnitude (treatment-speed interaction: HPC (lesion estimate): −0.182 ± 0.073 a.u./(cm/s), *t*_(16)_ = −2.488, χ^2^ = 11.189, *p* = 0.004; RSC (lesion estimate): −0.235 ± 0.062 a.u./(cm/s), *t*_(16)_ = −3.788, χ^2^ = 16.870, *p* < 0.001; Fig. 4*F*). Theta-band coherence, between HPC and RSC, increases with running speed and, consistent with the relationship between theta power and running speed, plateaus at higher speeds (>30 cm/s). Absolute coherence between RSC and HPC in MTT lesion animals was significantly increased compared across all speed bins (lesion estimate: 0.15 ± 0.05 a.u./(cm/s), *t*_(10)_ = 2.99, *p* = 0.017). In addition, the quadratic relationship between coherence and speed was attenuated in rats with MTT lesions (speed-treatment interaction: −5.46 × 10^−5^ ± 1.85 × 10^−5^ a.u./(cm/s), *t*_(10)_ = −2.96, χ^2^ = 9.24, *p* = 0.010; Fig. 4*G*).

During locomotion, gamma (30–90 Hz) power and frequency (Ahmed and Mehta, 2012) as well as coupling between theta phase and gamma amplitude (Sheremet et al., 2019) are positively correlated with running speed. Hippocampal gamma is thought to reflect the local interaction between inhibition and excitation generated from the interaction between pyramidal neurons and interneurons. Considering the effects observed in the theta frequency band, we next examined gamma (30–90 Hz) and HFOs (>90 Hz). Looking at coupling between the phase of theta and the amplitude of gamma oscillations, we first established, consistent with previous results (e.g., Belluscio et al., 2012; Koike et al., 2017), that gamma oscillations are dominant on the descending slope of the theta cycle, whereas RSC gamma is dominant closer to the theta peak (Fig. 4*H*). Using the time points of the troughs of gamma oscillations (>2 SDs of the baseline power) to generate the average of broadband filtered (0–500 Hz) LFP, we measured the time lag between the theta peak and the gamma trough (Fig. 4*H*) and found no difference between treatment groups. While temporal coupling appeared to be maintained in both regions, both the MI, derived from the phase amplitude coupling, as well as the gamma/HFO (30–200 Hz) of modulation, were significantly increased in the RSC of lesioned animals (Fig. 4*I–K*). This finding was associated with an increase in high-frequency power centered at ~170 Hz. (Fig. 4*K*). To determine whether the increased activity within this frequency range could be related to awake, cortical ripple-like activity, we analyzed 90–240 Hz power spectra across broad (10 cm/s) speed bins. Given that waking ripples typically occur during periods of wakeful immobility, the absence of ripple activity during active locomotion would discount this possibility. We found that HFO activity was present consistently across the speed range of lesioned animals, making it unlikely that the HFO (90–200 Hz), or the phase-amplitude coupling within this range was ripple-dependent. It is noteworthy that such HFOs (i.e., >90 Hz) may not reflect true oscillatory activity but instead may, in part, be influenced by multiunit activity around the electrode tip (Ray and Maunsell, 2011; Merker, 2013; Scheffer-Teixeira et al., 2013; Scheffer-Teixeira and Tort, 2017). Therefore, in as much as these effects may be classified as inhibitory/excitatory-related oscillatory imbalance, they may equally reflect local high-frequency hyperactivity.

#### MTT lesions reduce running speed-related theta asymmetry

**Figure 5.**
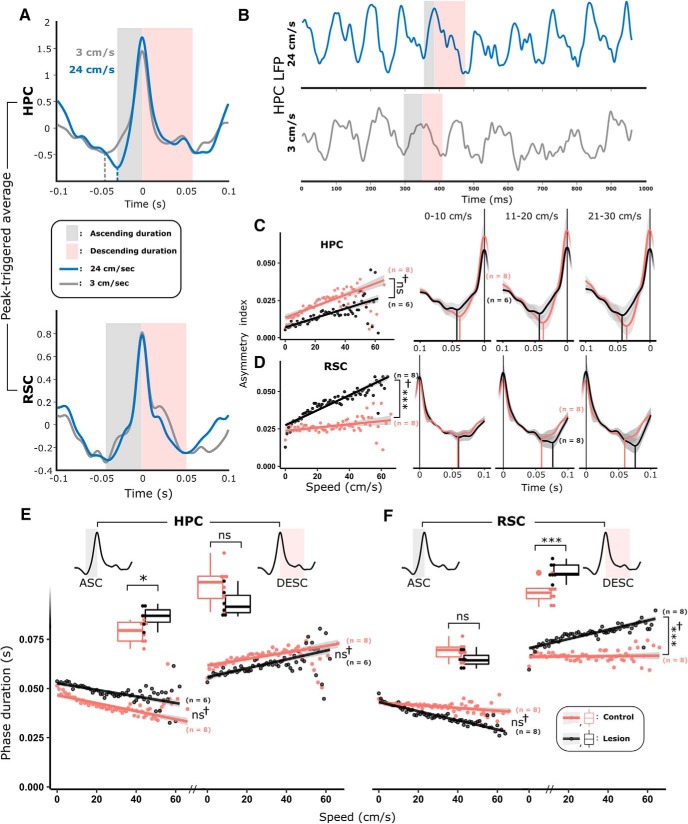
The hippocampal (HPC) theta cycle is an asymmetric, sawtooth waveform with a short ascending phase and a longer duration descending phase (***B***). Cycle asymmetry becomes exaggerated with increased rates of locomotion (***A–C***). The absolute asymmetry (asymmetry index [AI]) in the HPC theta cycle is highly correlated with running speed (***D***), reflecting an attenuation in ascending phase duration (***E***,***F***) and an increase in descending phase duration (***E***). Consistent with their observed peak frequency attenuation, MTT animals exhibited longer duration ascending phases than controls (***E***, left). Control RSC ascending phases decrease in duration with increasing speed (***F***) with consistent duration descending phases. In the RSC of MTT animals, cycle asymmetry is significantly more positively correlated with speed than in controls, resulting primarily from an increase in descending phase duration (***C***, bottom; ***F***). ***C–F***, Red represents control. Black represents lesion. *p < 0.05; ***p < 0.001; ns, not significant; ^†^Results of speed/treatment interactions.

To better understand the nature of the observed attenuation in theta frequency in MTT-lesion animals, we next looked at the dynamics of the theta cycle. Hippocampal theta cycles have an inverse saw-toothed shape, typically with a shorter duration ascending phase (trough-to-peak) and a longer descending phase (peak-to-trough), resulting in an asymmetric waveform (Buzsáki et al., 1985) (Fig. 5*A*,*B*). In controls, the asymmetry index (absolute difference in the duration of ascending and descending phases) within HPC theta oscillations was found to be positively correlated with running speed (control estimate): 0.46 ± 0.25 ms/(cm/s), *t*_(7)_ = 18.53, χ^2^ = 241.24, *p* < 0.001; Fig. 5*C*, top), resulting principally from a decrease in ascending phase duration at higher speeds (control estimate: −0.25 ± 0.13 ms/(cm/s), *t*_(7)_ = −19.73, *p* < 0.001; Fig. 5*E*). Consistent with our finding that MTT-lesion animals showed an intact speed/peak theta frequency relationship (Fig. 4), HPC theta ascending phases in lesioned animals were also significantly correlated with speed; however, ascending phase durations across all speed bins were significantly longer than controls (lesion estimate: 6.72 ± 2.53 ms, *t*_(13)_ = 2.66, *p* = 0.021; Fig. 5*C*,*E*).

In control animals, the strength of linear correlation between asymmetry index and running speed was considerably weaker in RSC than in HPC, but it was statistically significant (control estimate: 0.12 ± 0.027 ms/(cm/s), *t*_(7)_ = 4.35, χ^2^ = 18.46, *p* < 0.001). We found, however, that in lesioned animals, the RSC asymmetry index showed a considerably stronger increase with speed compared with control animals (treatment-speed interaction (lesion estimate): 0.37 ± 0.034 ms/(cm/s), *t*_(15)_ = 10.64, χ^2^ = 105.41, *p* < 0.001; Fig. 5*D*). This mainly arose from a greater increase in descending phase duration with speed (treatment × speed interaction: 0.23 ± 0.02 ms/(cm/s), *t*_(15)_ = 10.13, χ^2^ = 96.18, *p* < 0.001; Fig. 5*F*) and a greater attenuation in ascending phase duration in the lesion animals (treatment-speed interaction (lesion estimate): −0.16 ± 0.02 ms/(cm/s), *t*_(15)_ = −8.70, χ^2^ = 72.15, *p* < 0.001). Phase entrainment of RSC interneurons is thought to be independent of hippocampal input (Talk et al., 2004). Recent modeling of the dynamics of spike-phase coupling in the context of theta cycle asymmetry (Cole and Voytek, 2018) presents an interesting case that the duration of phase within a cycle is related to the firing frequency of phase-locked neurons such that longer duration descending phases in RSC may provide for a longer window in which firing activity may reach peak frequency, thus resulting in increased frequency activity. In the context of our findings, such a mechanism may serve to link the lesion-related changes in descending phase duration in RSC (Fig. 5*F*, right) with the observed increase in 180–240 Hz HFO (Fig. 4*I*, right).

#### MTT lesions attenuate theta frequency during PS

**Figure 6.**
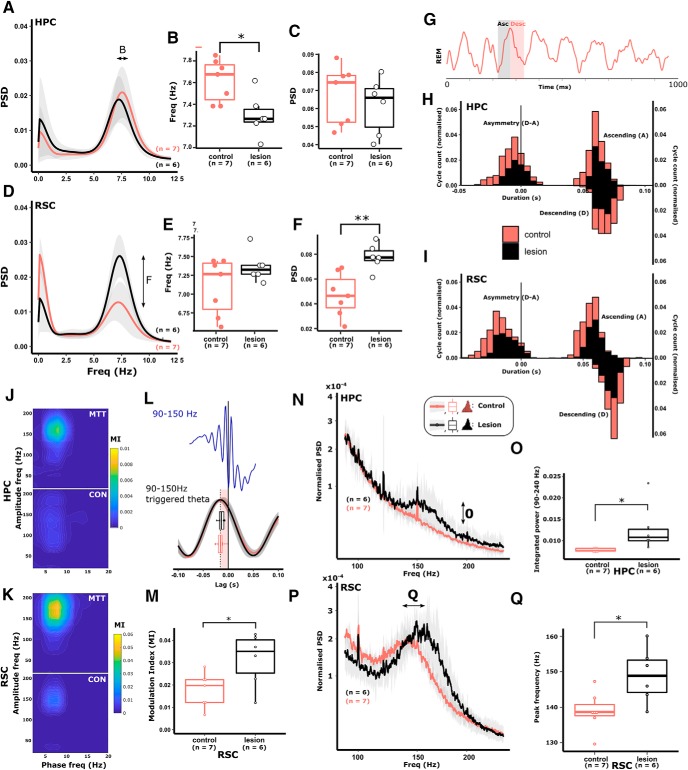
During PS, hippocampal theta frequency, but not power, was significantly attenuated (***A–C***), whereas cortical theta power, but not frequency, was significantly greater (***D–F***). ***G***, A typical LFP trace from HPC during PS. Shaded gray represents ascending phase. Shaded red represents descending phase. ***H***, ***I***, Lesion of the MTT did not influence the absolute asymmetry of theta cycles in either the HPC (***H***; left histogram) or the RSC (***I***; left histogram) as reflected by consistent, overlapping distributions of ascending and descending phase durations in both HPC and RSC (right histograms in ***H*** and ***I***, respectively). During PS, strong theta-gamma phase-amplitude coupling is present in the fast gamma frequency range (90–150 Hz; ***J***). The degree to which theta and fast gamma are coupled (MI) was significantly increased in both HPC (***J***) and RSC in lesioned animals (***K***, ***M***), whereas the time lag between the peak of the theta cycles, and the troughs of fast-gamma oscillations (>2 SDs of the baseline power) remained consistent between treatment groups (***L***). ***L***, The average theta waveform triggered by the trough of fast-gamma (90–150 Hz) oscillations (time = 0 s). Shaded red area represents the time lag between the trough of the gamma oscillations and the peak of the preceding theta peak, with corresponding box plots representing the very similar means, and variance of time lags compared between lesion (black) and control (red) animals. Lesion-dependent increases in phase-amplitude coupling in both HPC (***N***, ***O***) and RSC (***P***, ***Q***) were therefore the product of increased high-frequency power at frequencies that corresponded to the coupling observed in ***J*** and ***K***. Color key in ***N*** applies to all graphs in the figure: Red represents control. Black represents lesion. *p < 0.05; **p < 0.01.

To establish whether the lesion-related group effects on theta during locomotion were consistent across other high-theta (6–12 Hz) states, LFP recordings were made in the HPC and the RSC during paradoxical (REM) sleep (PS). During periods of PS, MTT lesion rats did not differ from controls in HPC theta power, however, a significant attenuation in theta frequency was apparent in lesioned animals (lesion estimate: −0.32 ± 0.11 Hz, *t*_(12)_ = −2.96, *p* = 0.013; Fig. 6*A–C*). In the RSC, lesioned animals had significantly higher theta power but no discernible difference in peak theta frequency (Fig. 6*D–F*). Theta cycles during PS were more symmetrical than during awake locomotion (Fig. 6*G*), and no difference between lesion and control groups was found in either the asymmetry index or ascending/descending phase durations in either HPC (Fig. 6*H*) or RSC (Fig. 6*I*).

During PS, RSC theta phase is strongly coupled to the amplitude of fast gamma (50–90 Hz)/HFO (90–200 Hz) amplitude (Koike et al., 2017) (Fig. 4*J*), with weaker phase amplitude coupling in HPC. The MI is a quantification of the strength (nonuniformity) of coupling between theta phase and the amplitude of gamma/HFO (Tort et al., 2010). Consistent with these findings, control animals showed strong theta-gamma/HFO phase-amplitude coupling during PS in RSC (Fig. 5*K*). Lesion animals were found to have a significant increase in MI during PS, centered around 170 Hz, in both HPC (control estimate: 0.01 ± 0.00, *t*_(12)_ = 2.45, *p* = 0.044; Fig. 6*J*) and RSC (control estimate: 0.01 ± 0.00, *t*_(12)_ = 2.23, *p* = 0.048; Fig. 6*K*,*M*). Consistent with the understanding that theta-slow gamma phase amplitude coupling is not differentially modulated within the sleep–wake cycle (Scheffer-Teixeira and Tort, 2017), no significant difference was found between treatment groups in either the MI or peak slow gamma amplitude frequency of the MI within the slow gamma frequency band (30–50 Hz).

In the RSC, the amplitude of gamma oscillations/HFO (30–150 Hz) is strongly coupled with the descending phase of the theta cycle during PS (Belluscio et al., 2012; Koike et al., 2017). To determine whether the phase (within the theta cycle) in which fast gamma was dominant was affected by disconnection of the MBs, we calculated the time lag between the trough of fast gamma/HFO oscillations (90–150 Hz; of power >2 SDs of the baseline), and the peak of the synchronous theta cycle. No significant group difference was observed in the mean lag time (Fig. 6*L*), suggesting that theta-entrainment of cortical unit activity was unaffected by MTT lesions; this is in line with evidence that RSC entrainment to theta is independent of HPC input (Talk et al., 2004). To test whether the increase in phase-amplitude coupling was related to nonuniformity associated with higher fast gamma/HFO power in MTT animals, we looked at the power spectral density within the frequency band in which phase-amplitude coupling peaked (90–240 Hz). Within this fast-gamma/HFO band, overall power was significantly higher in the HPC of lesioned animals compared with controls (0.004 ± 0.002, *t*_(12)_ = 2.375, *p* = 0.037; Fig. 6*N*,*O*). In the RSC of lesioned animals, the overall power within the 90–240 Hz band was not significantly increased, but the peak frequency of oscillations was significantly greater (lesion estimate: 10.083 ± 3.641 Hz, *t*_(12)_ = 3.641, *p* = 0.018; Fig. 6*P*,*Q*).

### Experiment 2: hippocampal spine and DCX analysis

#### MTT lesions reduce the number and clustering of CA1 spines and the number and complexity of DCX-positive neurons in dentate gyrus

**Figure 7.**
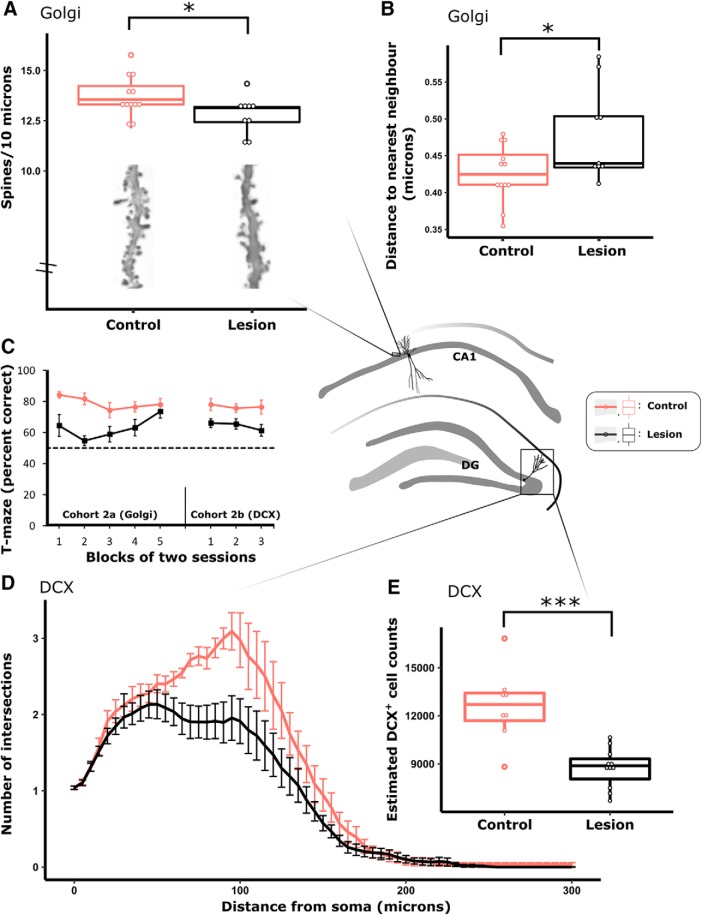
MTT lesions were associated with a decrease in both number (***A***) and clustering (***B***) of spines in the basal dendrites of Golgi-stained CA1 pyramidal neurons in Cohort 2a. Performance on a T-maze task was impaired in MTT lesion animals relative to controls in Cohort 2a and Cohort 2b (***C***). DCX is a marker of adult neurogenesis; in Cohort 2b, MTT lesions resulted in a decrease in the complexity of DCX^+^ neurons (***D***) as well as a decrease in the stereologically estimated overall number of DCX^+^ neurons in the dentate gyrus (DG; ***E***). Cohort 2a (lesion, *n* = 9; controls, *n* = 12); Cohort 2b (lesion, *n* = 12; control, *n* = 8). Central schematic diagram represents the morphology and anatomical location of neurons that were sampled. *p < 0.05; ***p < 0.001.

Since oscillatory activity and learning-induced plasticity are tightly coupled (e.g., Orr et al., 2001; Tsanov and Manahan-Vaughan, 2009), we hypothesized that changes in LFP might reflect altered structural plasticity in the hippocampus. MTT lesions have previously been shown to reduce markers of activity (e.g., c-Fos, Zif268, and cytochrome oxidase in RSC and c-Fos expression in HPC) (Vann and Albasser, 2009; Vann, 2013; Frizzati et al., 2016), which could reflect underlying microstructural changes. To address this possibility, we examined dendritic spine density and clustering in Golgi-stained tissue in one group of rats (Cohort 2a) and the expression of DCX, a marker of adult neurogenesis (Couillard-Despres et al., 2005), in a second group (Cohort 2b). Both groups underwent testing on a T-maze task (Dudchenko, 2001) and, consistent with previous studies (Vann, 2013; Nelson and Vann, 2014), the MTT lesion animals in both groups made more errors relative to controls (both *p* < 0.01; block main effect Cohort 2a, *F*_(4,76)_ = 1.77, *p* = 0.14; Cohort 2b, F < 1; interaction Cohort 2a, *F*_(4,76)_ = 1.94, *p* = 0.11; Cohort 2b, *F* < 1). When comparing overall performance across the blocks in each cohort, both MTT lesion and control animals' performance was above chance (Cohort 2a: lesion, *p* < 0.05; control, *p* < 0.01; Cohort 2b, both *p* < 0.01; Fig. 7*C*).

MTT lesions reduced the density of spines on basal CA1 segments (*t*_(19)_ = 2.23, *p* = 0.038; Fig. 7*A*). Furthermore, the distribution of CA1 spine clustering was significantly attenuated in lesioned compared with control animals (*t*_(19)_ = 2.37, *p* = 0.028; Fig. 7*B*). In contrast to the changes in CA1, there were no lesion-related changes in either the density (*t*_(19)_ = 0.38, *p* = 0.71) or clustered distribution (Mann–Whitney *U* = 42.00, *p* = 0.39) of spines in the apical segments of RSC pyramidal neurons. Both spine density and clustering are associated with learning and memory in intact animals (Moser et al., 1994, 1997; Harland et al., 2014); spine clustering is thought to underlie stronger and more efficient information processing in dendrites (Rogerson et al., 2014; Kastellakis et al., 2015). The reduction in CA1 spine density and clustering in the lesion animals may therefore be an additional contributor to impoverished encoding in this lesion model.

Spatial training has been linked to structural changes within existing hippocampal cells but also to increased numbers and dendritic arbor complexity of adult-born neurons in the dentate gyrus (Kempermann et al., 2004; Vukovic et al., 2013). MTT lesions reduced the unbiased stereological estimation of the number of dentate gyrus DCX cells by ~31% (*t*_(17)_ = 4.76, *p* < 0.001; Fig. 7*E*). In addition to there being fewer DCX cells in the lesion group, those cells that were present exhibited less morphological complexity, as reflected by a reduced number of intersections (Sholl analyses; *F*_(1,18)_ = 9.05, *p* = 0.008; radius main effect, *p* < 0.001; interaction, *p* = 0.07; Fig. 7*D*). In contrast, MTT lesions did not influence the number of intersections for either dentate gyrus or CA1 basal Golgi-stained hippocampal arbors (both *F* < 1; radius main effect both *p* < 0.01; interaction both *F* < 1) or Golgi-stained RSC basal or apical arbors (basal, *F* < 1; apical, *F*_(1,19)_ = 1.67, *p* = 0.21; radius main effect both *p* < 0.01; interaction both *F* < 1). Therefore, differences in morphological complexity appear limited to newly formed hippocampal cells. The reduction in DCX cells may be a contributing factor to the memory impairments associated with MTT lesion as targeted experimental suppression of hippocampal neurogenesis impairs performance on spatial memory tasks (Dupret et al., 2008; Farioli-Vecchioli et al., 2008; Lemaire et al., 2012).

### Experiment 3: MR diffusivity

#### Diffusion-weighted imaging and microstructural plastic changes

The reductions in DCX-positive cells and CA1 spine density were observed following training on a spatial memory task. We therefore hypothesized that MTT lesions led to the attenuation of experience-driven structural plasticity and tested this prediction by using a longitudinal MRI study. Spatial learning has been found to evoke changes in gray matter diffusivity in the hippocampal formation of both humans and rodents. Moreover, in one study, altered diffusivity co-occurred with enhanced staining for various plasticity markers, such as synaptic boutons, glial reactivity, and BDNF levels (Sagi et al., 2012; Tavor et al., 2013). Diffusion-weighted MRI therefore offers a noninvasive means of investigating correlates of experience-driven plastic changes in intact and lesion animals.

#### Brainwide lesion-induced diffusivity changes

**Figure 8.**
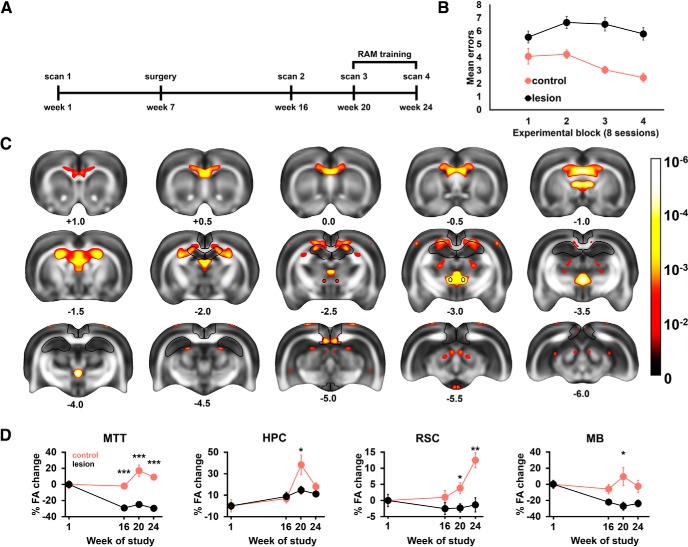
Animals were scanned at four time points over a 6 month period (***A***); surgeries were performed during week 7. MTT lesion rats (*n* = 13; controls, *n* = 7) were impaired on a radial-arm maze task (***B***). Changes in FA elicited by MTT lesion and radial-arm maze training are shown as results of an ANOVA with the between-factor of surgery (two levels) and the within-factor of scan number (four levels). Significant voxels have been thresholded to α < 0.01 and displayed using a heat map overlaid on top of a mean FA template (***C***). Successive coronal levels are numbered according to distance from bregma (mm). Outlines designate anatomical structures from which ROIs for regional analyses were selected (at α < 0.005). ***C***, The heatmap calibration bar represents *p* values on a logarithmic scale (−Log_10_). Graphs showing the timeline of regional changes in the MTT, hippocampus (HPC), RSC, and the MBs with the *y* axis displaying changes from the mean baseline (prospective lesion and control cases) values at Scan 1 (***D***). Error bars indicate SEM. *p < 0.05; **p < 0.01; ***p < 0.001.

Rats (Cohort 3) were scanned at four separate time points: before surgery (Scan 1); nine weeks following MTT-lesion surgery or control surgery (Scan 2); at the beginning of radial-arm maze (RAM) training (Scan 3); and at completion of RAM training when the controls were proficient at the task (Scan 4; Fig. 8*A*,*B*). The MTT lesion animals were significantly impaired on the RAM task (*F*_(1,18)_ = 14.08, *p* = 0.001; Fig. 8*B*). We used a voxelwise mixed ANOVA to uncover brainwide interactions between group (lesion vs control) and scan time (four levels) for two complementary diffusivity metrics, FA and MD (Feldman et al., 2010). Since differential changes in MD appeared to derive mainly from the ventricular system (presumably a direct effect of ventricular enlargement following the lesion), this measure was not analyzed further. On the other hand, voxels where differences in FA values reached significance (*p* < 0.05) were found across many large white matter structures, such as the corpus callosum, cingulum, fornix, and the MTT (Fig. 8*C*). Clusters of significant voxels were also present in gray matter, including the hippocampus, MBs, posterior granular RSC, and parts of the midbrain.

#### Higher hippocampal and retrosplenial FA following spatial learning in intact animals, but not in lesion cases

We next performed regional *post hoc* comparisons to examine the timeline of diffusivity changes more closely. The selected regions of interest comprised clusters at α < 0.005 (uncorrected, see Materials and Methods) present in the anterior dorsal hippocampus (HPC), posterior granular RSC, posterolateral medial MBs and, for comparison, in the MTT (Fig. 7*D*).

Consistent with previous reports, spatial training elicited higher FA in intact animals (Sagi et al., 2012). While the MTT and HPC values peaked following initial training (Scan 1 vs Scan 3: MTT, *p* = 0.035; HPC, *p* < 0.001), the RSC values showed a gradual increase, reaching maximum levels following the final test session (*p* = 0.008 at Scan 3 and *p* < 0.001 at Scan 4). This complementary pattern of diffusivity changes is consistent with the medial diencephalon and hippocampus supporting memory encoding (Vann and Aggleton, 2003) with the RSC having a greater role in long-term memory storage (Milczarek et al., 2018). By contrast, lesion cases exhibited markedly attenuated FA in relation to initial training (MTT: *p* < 0.001, MB: *p* < 0.043, HPC: *p* = 0.011, RSC: *p* = 0.018) as well as following the final training session in the RSC (*p* = 0.001). While the first postsurgery scan (Scan 2; pretraining) revealed altered FA values in the MB and MTT (regions that have sustained primary and secondary damage; *p* < 0.001), the differences in HPC and RSC, sites distal to the lesion, were not observed until animals had undergone spatial training. We therefore interpret these distal changes in diffusivity as a failure to express task-evoked plasticity in MTT lesion animals.

## Discussion

The traditional view of the Papez circuit is that the MBs and anterior thalamic nuclei are functionally downstream of the hippocampus, acting principally as a hippocampal relay (Papez, 1937; Barbizet, 1963; Aggleton and Brown, 1999). Instead, the present results support a revised model whereby the MBs have an important role in optimizing hippocampal-cortical oscillations, in particular modulating the frequency of theta, the level of interregional coherence and, in turn, local hippocampal and cortical activity. Concordantly, MTT lesions diminish a number of markers of learning-induced plasticity, which likely contribute to the marked memory impairments observed.

MTT lesions were used to model diencephalic amnesia, enabling us to disentangle the contribution of the MBs without concomitant damage to the supramammillary nuclei. This is important given the influence of the supramammillary nuclei on hippocampal theta frequency (e.g., Kirk, 1998). As such, previous studies that have assessed the effects of MB lesions, or inactivation, on hippocampal theta can be difficult to interpret when damage extends into the overlying supramammillary nuclei (Sharp and Koester, 2008; Zakowski et al., 2017), and vice versa (Renouard et al., 2015). Nevertheless, the only previous study that has examined hippocampal theta following MB manipulation in nonanesthetized animals established a similar pattern of changes to those reported here, with a reduction in hippocampal theta frequency (Sharp and Koester, 2008). The lesions in the Sharp and Koester (2008) study included both the MBs and supramammillary nuclei; however, our present findings would suggest that their results were at least in part driven by the loss of the MBs. Theta frequency is considered a mechanism for timing the flow of information in the hippocampus (Richard et al., 2013), and high-frequency theta in particular has been linked to spatial memory in both rodents and humans (Olvera-Cortés et al., 2002; Goyal et al., 2018). Furthermore, reducing the frequency of theta in otherwise normal animals impairs performance on spatial memory tasks (Pan and McNaughton, 1997). The implication, therefore, is that this reduction in hippocampal theta frequency would contribute to the memory impairment observed in MTT lesion animals.

In our revised model of the MB contribution to the Papez circuit, the medial mammillary nuclei integrate theta input from the ventral tegmental nuclei of Gudden. Thus, to more fully characterize any dysfunction associated with disconnection of this theta stream, we assessed hippocampal-cortical activity across two theta-rich states: awake locomotion and PS. During locomotion, several characteristics of theta (e.g., frequency, power, cycle waveform characteristics) are correlated with running speed; and for the most part, these associations were spared by MB disconnection: that is, theta frequency was attenuated overall, but its relationship with running speed was preserved.

Not only was there a frequency attenuation in the lesion animals during locomotion, but the HPC theta cycle in these animals exhibited a more symmetrical waveform, characterized by longer-duration ascending (trough-to-peak) phases. As in HPC, RSC theta asymmetry is positively correlated with running speed (albeit to a lesser degree than in HPC). An unexpected finding of the study was that the RSC descending phase duration of lesioned animals increased dramatically with running speed relative to controls (Fig. 5*F*). This change was accompanied by a large increase in HFO (180–240 Hz) power and significantly higher phase-amplitude coupling at a frequency corresponding to the spectral peak of the HFO (Fig. 8*F*,*G*). Inhibitory interneurons fire preferentially in the descending phase of the theta cycle (Buzsáki, 2002). Extrapolating from the findings in the HPC of Cole and Voytek (2018), this pattern of effects may be explained by relating the putative RSC hyperactivity to the observed changes in the dynamics of the theta cycle such that, with longer descending phases, phase-locked interneurons have an increased “preferred-phase window” within which to reach their intrinsic firing frequency, resulting in a greater maximally attained firing frequency across each cycle.

**Figure 9.**
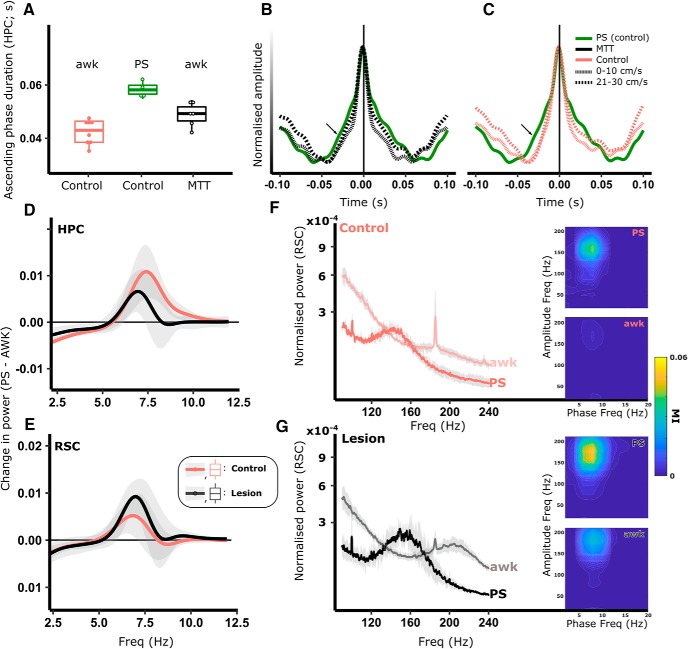
Both active locomotion and PS are “theta-rich” states. Theta cycles in PS are more symmetrical (***A–C***) than during locomotion (AWK). In animals with lesions of the MTT, theta cycles during locomotion were closer in symmetry to PS cycles than those of controls (***A–C***). In the hippocampus (HPC) of lesioned animals, the frequency of theta oscillations was reduced during both AWK and PS, which is highlighted by the within-recording subtraction of the AWK power spectrum from that of PS (***D***). While theta frequency was reduced in RSC during AWK, it was not reduced during PS (***E***); however, theta power was significantly increased in lesion animals (***E***; PS power spectrum − AWK power spectrum). The observed increase in RSC power during PS was accompanied by an increase in the power and frequency of activity in the 100–180 Hz range (HFA; ***F***, ***G***), as well as an increase in phase amplitude coupling between theta and HFA (PS MI heatmaps in ***F***, ***G***). Increased phase-amplitude coupling present in the RSC during AWK (AWK MI heatmaps in ***F***, ***G***) was found to be due to a peak in HFA, but at a considerably higher frequency (180–240 Hz) than that found during PS, reinforcing the likelihood that distinct mechanisms, via independent neural populations, are responsible for the observed changes in awake locomotion and PS.

In control animals, hippocampal theta cycles during PS are more symmetrical than during locomotion (Fig. 9*A–C*). As a result, longer ascending phase durations of awake locomoting lesioned animals were found to be closer to control PS cycles than those of control locomoting animals (Fig. 9*A*), suggesting that, in lesioned animals, theta cycles are less able to vary between states. Silencing septal theta during PS dramatically attenuates hippocampal theta power and disrupts contextual memory (Boyce et al., 2016); although MB disconnection did not disrupt hippocampal theta to the same degree, it did lead to an attenuation in theta frequency during PS, potentially highlighting a previously unexplored involvement of these structures in consolidation. While HPC theta frequency was reduced in the lesion animals both during locomotion and PS, the RSC showed some state-dependent changes in theta. In contrast to the reduced theta frequency during locomotion, the frequency of PS theta in the RSC was not reduced; and instead, a significant increase in theta power was observed (Fig. 6*D*,*F*). The increased HFO in the RSC observed during locomotion in MTT lesion animals was also found during PS (Fig. 9*G*); but given that the peak frequency of HFO during PS was considerably lower than during locomotion, it is possible that these changes may reflect activity changes in separate subpopulations of the RSC that are specifically active during either PS (68% of RSC units reported by Koike et al., 2017) or during locomotion. Unlike in awake animals, however, no effect was seen in waveform dynamics of the theta cycle, leaving open an explanation for the HFO changes in PS in both HPC (Fig. 6*N*,*O*) and RSC (Fig. 9). One mechanism by which MB projections could influence the frequency of hippocampocortical theta frequency is through the cholinergic system. Following chemogenetic inhibition of cholinergic neurons in the medial septum, Carpenter et al. (2017) reported an attenuation in theta frequency in the hippocampal formation that mirrored the magnitude and direction of changes reported here following MTT lesions. Both hippocampal and cortical cholinergic activity is reduced following MB lesions (Béracochéa et al., 1995); although the mammillothalamic projection itself is not cholinergic, it may act to modulate cholinergic activity through interactions either with its direct connections with the anteroventral thalamic nucleus/nucleus basalis of Meynert, or through indirect influence on distal brain regions. One such pathway, described by Savage et al. (2011) in explanation of reduced cholinergic activity in HPC following lesions of the anterior thalamus, is via the modulation of descending RSC projections to the medial septum (Gonzalo-Ruiz and Morte, 2000).

Possible lesion-induced hyperactivity in RSC (Fig. 8) may reflect a more synchronized (i.e., deactivated) cortical state in which there is a reduction in the variance of population-wide activity (Harris and Thiele, 2011). Both the observed increase in theta power during PS, the corresponding increase in HFO, as well as conserved theta phase coupling, support a case for increased local synchronicity. Animals with MTT lesions also exhibited excessive interregional coherence across both awake and PS states, which again likely reflects a reduction in functional diversity in the system following the loss of ascending MB projections. Indeed, such excessive synchrony is thought to reduce information coding capacity (Cagnan et al., 2015), whereas increased regional connectivity has been observed in neurological conditions linked to impaired cognitive ability (Hawellek et al., 2011; Gardini et al., 2015).

Several distal microstructural differences were also observed in MTT-lesioned rats, with fewer spines present on the basal dendrites of CA1 pyramidal neurons, and a corresponding reduction in spine clustering (Fig. 7); the latter a mechanism that, in normal animals, likely serves to strengthen neural responses (Kastellakis et al., 2015). Rats with MTT lesions had significantly fewer DCX-positive neurons in the dentate gyrus, and those that were present showed a reduced morphological complexity, suggesting that mammillothalamic disconnection may be associated with a reduction in adult hippocampal neurogenesis. Given that all of these measures increase in normal animals following spatial training (Moser et al., 1997; Tronel et al., 2010; Lemaire et al., 2012; Vukovic et al., 2013), this combination of observed microstructural differences in lesion animals may well reflect a reduction in the capacity for learning-induced plasticity. Such an explanation is further supported by our diffusion-MR study where the distal impact of lesioning the MTT became evident only once animals underwent spatial training, suggesting the loss of learning-induced plasticity in lesion cases. Together, these convergent findings reinforce the pervasive effects of MTT lesions on numerous aspects of neural plasticity.

There is increasing evidence of mammillothalamic pathology in neurological conditions that present with memory impairments (Kumar et al., 2008, 2009; e.g., Denby et al., 2009; Dzieciol et al., 2017; Perry et al., 2019), reinforcing the need to better understand the contributions of these medial diencephalic structures. Together, our results provide insight into mechanisms via which the MBs can contribute to memory processes: ascending mammillary projections indirectly optimize hippocampocortical plasticity via their contribution to oscillatory architecture. Furthermore, given the changes observed during PS, it is possible that this region is not only involved in encoding, as previously assumed (Vann and Nelson, 2015), but may also have additional roles in memory consolidation. If distal oscillatory disturbances underlie the memory impairments following medial diencephalic damage, it raises the possibility that artificially restoring the electrophysiological patterns to normal could serve as a therapeutic intervention. Deep brain stimulation of the hypothalamus, fornix, and medial temporal lobe has been reported to improve memory function and increase plasticity and neurogenesis (Lee et al., 2013); the current findings provide a possible mechanism via which these memory improvements may occur.

## References

[B1] Aggleton JP, Brown MW (1999) Episodic memory, amnesia, and the hippocampal-anterior thalamic axis. Behav Brain Sci 22:425–444; discussion 444–489. 11301518

[B2] Ahmed OJ, Mehta MR (2012) Running speed alters the frequency of hippocampal gamma oscillations. J Neurosci 32:7373–7383. 10.1523/JNEUROSCI.5110-11.2012 22623683 PMC3366345

[B3] Avants BB, Epstein CL, Grossman M, Gee JC (2008) Symmetric diffeomorphic image registration with cross-correlation: evaluating automated labeling of elderly and neurodegenerative brain. Med Image Anal 12:26–41. 10.1016/j.media.2007.06.004 17659998 PMC2276735

[B4] Avants BB, Tustison NJ, Song G, Cook PA, Klein A, Gee JC (2011) A reproducible evaluation of ANTs similarity metric performance in brain image registration. Neuroimage 54:2033–2044. 10.1016/j.neuroimage.2010.09.025 20851191 PMC3065962

[B5] Barbizet J (1963) Defect of memorizing of hippocampal-mammillary origin: a review. J Neurol Neurosurg Psychiatry 26:127–135. 10.1136/jnnp.26.2.127 13966551 PMC495548

[B6] Bates D, Mächler M, Bolker B, Walker S (2015) Fitting Linear Mixed-Effects Models Using lme4. Journal of Statistical Software 67:1–48.

[B7] Belluscio MA, Mizuseki K, Schmidt R, Kempter R, Buzsáki G (2012) Cross-frequency phase-phase coupling between theta and gamma oscillations in the hippocampus. J Neurosci 32:423–435. 10.1523/JNEUROSCI.4122-11.2012 22238079 PMC3293373

[B8] Béracochéa DJ, Micheau J, Jaffard R (1995) Alteration of cortical and hippocampal cholinergic activities following lesion of the mammillary bodies in mice. Brain Res 670:53–58. 10.1016/0006-8993(94)01253-E 7719724

[B9] Bikbaev A, Manahan-Vaughan D (2008) Relationship of hippocampal theta and gamma oscillations to potentiation of synaptic transmission. Front Neurosci 2:56–63. 10.3389/neuro.01.010.2008 18982107 PMC2570077

[B10] Boyce R, Glasgow SD, Williams S, Adamantidis A (2016) Causal evidence for the role of REM sleep theta rhythm in contextual memory consolidation. Science 352:812–816. 10.1126/science.aad5252 27174984

[B11] Briess D, Cotter D, Doshi R, Everall I (1998) Mamillary body abnormalities in schizophrenia. Lancet 352:789–790. 10.1016/S0140-6736(05)60685-x 9737293

[B12] Buzsáki G (2002) Theta oscillations in the hippocampus. Neuron 33:325–340. 10.1016/S0896-6273(02)00586-X 11832222

[B13] Buzsáki G, Rappelsberger P, Kellényi L (1985) Depth profiles of hippocampal rhythmic slow activity ('theta rhythm') depend on behaviour. Electroencephalogr Clin Neurophysiol 61:77–88. 10.1016/0013-4694(85)91075-2 2408867

[B14] Cagnan H, Duff EP, Brown P (2015) The relative phases of basal ganglia activities dynamically shape effective connectivity in Parkinson's disease. Brain 138:1667–1678. 10.1093/brain/awv093 25888552 PMC4614137

[B15] Canolty RT, Knight RT (2010) The functional role of cross-frequency coupling. Trends Cogn Sci 14:506–515. 10.1016/j.tics.2010.09.001 20932795 PMC3359652

[B16] Carlesimo GA, Serra L, Fadda L, Cherubini A, Bozzali M, Caltagirone C (2007) Bilateral damage to the mammillo-thalamic tract impairs recollection but not familiarity in the recognition process: a single case investigation. Neuropsychologia 45:2467–2479. 10.1016/j.neuropsychologia.2007.03.025 17512561

[B17] Carlesimo GA, Lombardi MG, Caltagirone C (2011) Vascular thalamic amnesia: a reappraisal. Neuropsychologia 49:777–789. 10.1016/j.neuropsychologia.2011.01.026 21255590

[B18] Carpenter F, Burgess N, Barry C (2017) Modulating medial septal cholinergic activity reduces medial entorhinal theta frequency without affecting speed or grid coding. Sci Rep 7:14573. 10.1038/s41598-017-15100-6 29109512 PMC5673944

[B19] Chang LC, Jones DK, Pierpaoli C (2005) RESTORE: robust estimation of tensors by outlier rejection. Magn Reson Med 53:1088–1095. 10.1002/mrm.20426 15844157

[B20] Clarke S, Assal G, Bogousslavsky J, Regli F, Townsend DW, Leenders KL, Blecic S (1994) Pure amnesia after unilateral left polar thalamic infarct: topographic and sequential neuropsychological and metabolic (PET) correlations. J Neurol Neurosurg Psychiatry 57:27–34. 10.1136/jnnp.57.1.27 8301301 PMC485036

[B21] Cole SR, Voytek B (2018) Hippocampal theta bursting and waveform shape reflect CA1 spiking patterns. bioRxiv. Advance online publication. Retrieved October 25, 2018. doi:10.1101/452987 10.1101/452987

[B22] Couillard-Despres S, Winner B, Schaubeck S, Aigner R, Vroemen M, Weidner N, Bogdahn U, Winkler J, Kuhn HG, Aigner L (2005) Doublecortin expression levels in adult brain reflect neurogenesis. Eur J Neurosci 21:1–14. 10.1111/j.1460-9568.2004.03813.x 15654838

[B23] Delay J, Brion S (1969) Le Syndrome de Korsakoff. Paris: Masson.

[B24] Denby CE, Vann SD, Tsivilis D, Aggleton JP, Montaldi D, Roberts N, Mayes AR (2009) The frequency and extent of mammillary body atrophy associated with surgical removal of a colloid cyst. AJNR Am J Neuroradiol 30:736–743. 10.3174/ajnr.A1424 19164441 PMC7051749

[B25] Dillingham CM, Frizzati A, Nelson AJ, Vann SD (2015) How do mammillary body inputs contribute to anterior thalamic function? Neurosci Biobehav Rev 54:108–119. 10.1016/j.neubiorev.2014.07.025 25107491 PMC4462591

[B26] Dorph-Petersen KA, Nyengaard JR, Gundersen HJ (2001) Tissue shrinkage and unbiased stereological estimation of particle number and size. J Microsc 204:232–246. 10.1046/j.1365-2818.2001.00958.x 11903800

[B27] Dudchenko PA (2001) How do animals actually solve the T maze? Behav Neurosci 115:850–860. 10.1037/0735-7044.115.4.850 11508724

[B28] Dupret D, Revest JM, Koehl M, Ichas F, De Giorgi F, Costet P, Abrous DN, Piazza PV (2008) Spatial relational memory requires hippocampal adult neurogenesis. PLoS One 3:e1959. 10.1371/journal.pone.0001959 18509506 PMC2396793

[B29] Dzieciol AM, Bachevalier J, Saleem KS, Gadian DG, Saunders R, Chong WK, Banks T, Mishkin M, Vargha-Khadem F (2017) Hippocampal and diencephalic pathology in developmental amnesia. Cortex 86:33–44. 10.1016/j.cortex.2016.09.016 27880886 PMC5264402

[B30] Farioli-Vecchioli S, Saraulli D, Costanzi M, Pacioni S, Cinà I, Aceti M, Micheli L, Bacci A, Cestari V, Tirone F (2008) The timing of differentiation of adult hippocampal neurons is crucial for spatial memory. PLoS Biol 6:e246. 10.1371/journal.pbio.0060246 18842068 PMC2561078

[B31] Feldman HM, Yeatman JD, Lee ES, Barde LH, Gaman-Bean S (2010) Diffusion tensor imaging: a review for pediatric researchers and clinicians. J Dev Behav Pediatr 31:346–356. 10.1097/DBP.0b013e3181dcaa8b 20453582 PMC4245082

[B32] Frizzati A, Milczarek MM, Sengpiel F, Thomas KL, Dillingham CM, Vann SD (2016) Comparable reduction in Zif268 levels and cytochrome oxidase activity in the retrosplenial cortex following mammillothalamic tract lesions. Neuroscience 330:39–49. 10.1016/j.neuroscience.2016.05.030 27233617 PMC4936792

[B33] Fuhrmann F, Justus D, Sosulina L, Kaneko H, Beutel T, Friedrichs D, Schoch S, Schwarz MK, Fuhrmann M, Remy S (2015) Locomotion, theta oscillations, and the speed-correlated firing of hippocampal neurons are controlled by a medial septal glutamatergic circuit. Neuron 86:1253–1264. 10.1016/j.neuron.2015.05.001 25982367

[B34] Gamper E (1928) Zur frage der polioencephalitis der chronischen alkoholiker. anatomische befunde beim chronischem korsakow und ihre beziehungen zum klinischen bild. Dtsch Z Nervenheilkd 102:122–129. 10.1007/BF01668327

[B35] Gardini S, Venneri A, Sambataro F, Cuetos F, Fasano F, Marchi M, Crisi G, Caffarra P (2015) Increased functional connectivity in the default mode network in mild cognitive impairment: a maladaptive compensatory mechanism associated with poor semantic memory performance. J Alzheimers Dis 45:457–470. 10.3233/JAD-142547 25547636

[B36] Gonzalo-Ruiz A, Morte L (2000) Localization of amino acids, neuropeptides and cholinergic markers in neurons of the septum-diagonal band complex projecting to the retrosplenial granular cortex of the rat. Brain Res Bull 52:499–510. 10.1016/S0361-9230(00)00287-2 10974489

[B37] Goyal A, Miller J, Qasim S, Watrous AJ, Stein JM, Inman CS, Gross RE, Willie JT, Lega B, Lin JJ, Sharan A, Wu C, Sperling MR, Sheth SA, McKhann GM, Smith EH, Schevon C, Jacobs J (2018) Functionally distinct high and low theta oscillations in the human hippocampus. bioRxiv. Advance online publication. Retrieved December 17, 2018. doi:10.1101/498055 10.1101/498055 PMC7235253 32424312

[B38] Gudden H (1896) Klinische und anatomische Beitrage zur Kenntnis der multiplen Alkoholneuritis nebst Bernerkungen uber die Regenerationsvorgange im peripheren Nervensystem. Arch Psychiatr 28:643–741.

[B39] Gundersen HJ (1986) Stereology of arbitrary particles: a review of unbiased number and size estimators and the presentation of some new ones, in memory of William R. Thompson. J Microsc 143:3–45. 10.1111/j.1365-2818.1986.tb02764.x 3761363

[B40] Gundersen HJ, Jensen EB, Kiêu K, Nielsen J (1999) The efficiency of systematic sampling in stereology–reconsidered. J Microsc 193:199–211. 10.1046/j.1365-2818.1999.00457.x 10348656

[B41] Harland BC, Collings DA, McNaughton N, Abraham WC, Dalrymple-Alford JC (2014) Anterior thalamic lesions reduce spine density in both hippocampal CA1 and retrosplenial cortex, but enrichment rescues CA1 spines only. Hippocampus 24:1232–1247. 10.1002/hipo.22309 24862603

[B42] Harris KD, Thiele A (2011) Cortical state and attention. Nat Rev Neurosci 12:509–523. 10.1038/nrn3084 21829219 PMC3324821

[B43] Hawellek DJ, Hipp JF, Lewis CM, Corbetta M, Engel AK (2011) Increased functional connectivity indicates the severity of cognitive impairment in multiple sclerosis. Proc Natl Acad Sci U S A 108:19066–19071. 10.1073/pnas.1110024108 22065778 PMC3223469

[B44] Huerta PT, Lisman JE (1993) Heightened synaptic plasticity of hippocampal CA1 neurons during a cholinergically induced rhythmic state. Nature 364:723–725. 10.1038/364723a0 8355787

[B45] Jankowski MM, Ronnqvist KC, Tsanov M, Vann SD, Wright NF, Erichsen JT, Aggleton JP, O'Mara SM (2013) The anterior thalamus provides a subcortical circuit supporting memory and spatial navigation. Front Syst Neurosci 7:45. 10.3389/fnsys.2013.00045 24009563 PMC3757326

[B46] Jeewajee A, Lever C, Burton S, O'Keefe J, Burgess N (2008) Environmental novelty is signaled by reduction of the hippocampal theta frequency. Hippocampus 18:340–348. 10.1002/hipo.20394 18081172 PMC2678674

[B47] Jones DK, Horsfield MA, Simmons A (1999) Optimal strategies for measuring diffusion in anisotropic systems by magnetic resonance imaging. Magn Reson Med 42:515–525. 10.1002/(SICI)1522-2594(199909)42:3<515::AID-MRM14>3.0.CO;2-Q 10467296

[B48] Kastellakis G, Cai DJ, Mednick SC, Silva AJ, Poirazi P (2015) Synaptic clustering within dendrites: an emerging theory of memory formation. Prog Neurobiol 126:19–35. 10.1016/j.pneurobio.2014.12.002 25576663 PMC4361279

[B49] Kempermann G, Jessberger S, Steiner B, Kronenberg G (2004) Milestones of neuronal development in the adult hippocampus. Trends Neurosci 27:447–452. 10.1016/j.tins.2004.05.013 15271491

[B50] Kikinis Z, Makris N, Sydnor VJ, Bouix S, Pasternak O, Coman IL, Antshel KM, Fremont W, Kubicki MR, Shenton ME, Kates WR, Rathi Y (2019) Abnormalities in gray matter microstructure in young adults with 22q11.2 deletion syndrome. Neuroimage Clin 21:101611. 10.1016/j.nicl.2018.101611 30522971 PMC6411601

[B51] Kirk IJ (1998) Frequency modulation of hippocampal theta by the supramammillary nucleus, and other hypothalamo-hippocampal interactions: mechanisms and functional implications. Neurosci Biobehav Rev 22:291–302. 10.1016/S0149-7634(97)00015-8 9579319

[B52] Kocsis B, Vertes RP (1994) Characterization of neurons of the supramammillary nucleus and mammillary body that discharge rhythmically with the hippocampal theta rhythm in the rat. J Neurosci 14:7040–7052. 10.1523/JNEUROSCI.14-11-07040.1994 7965097 PMC6577300

[B53] Koike BD, Farias KS, Billwiller F, Almeida-Filho D, Libourel PA, Tiran-Cappello A, Parmentier R, Blanco W, Ribeiro S, Luppi PH, Queiroz CM (2017) Electrophysiological evidence that the retrosplenial cortex displays a strong and specific activation phased with hippocampal theta during paradoxical (REM) sleep. J Neurosci 37:8003–8013. 10.1523/JNEUROSCI.0026-17.2017 28729438 PMC6596907

[B54] Kopelman MD (1995) The Korsakoff syndrome. Br J Psychiatry 166:154–173. 10.1192/bjp.166.2.154 7728359

[B55] Kumar R, Birrer BV, Macey PM, Woo MA, Gupta RK, Yan-Go FL, Harper RM (2008) Reduced mammillary body volume in patients with obstructive sleep apnea. Neurosci Lett 438:330–334. 10.1016/j.neulet.2008.04.071 18486338

[B56] Kumar R, Woo MA, Birrer BV, Macey PM, Fonarow GC, Hamilton MA, Harper RM (2009) Mammillary bodies and fornix fibers are injured in heart failure. Neurobiol Dis 33:236–242. 10.1016/j.nbd.2008.10.004 19022386 PMC2745998

[B57] Lee H, Fell J, Axmacher N (2013) Electrical engram: how deep brain stimulation affects memory. Trends Cogn Sci 17:574–584. 10.1016/j.tics.2013.09.002 24126128

[B58] Leemans A, Jones DK (2009) The B-matrix must be rotated when correcting for subject motion in DTI data. Magn Reson Med 61:1336–1349. 10.1002/mrm.21890 19319973

[B59] Leemans A, Jeurissen B, Sijbers J, Jones DK (2009) ExploreDTI: a graphical toolbox for processing, analyzing, and visualizing diffusion MR data. 17th Annual Meeting of International Society of Magnetic Resonance in Medicine, Honolulu.

[B60] Lemaire V, Tronel S, Montaron MF, Fabre A, Dugast E, Abrous DN (2012) Long-lasting plasticity of hippocampal adult-born neurons. J Neurosci 32:3101–3108. 10.1523/JNEUROSCI.4731-11.2012 22378883 PMC6622037

[B61] Leung LW, Lopes da Silva FH, Wadman WJ (1982) Spectral characteristics of the hippocampal EEG in the freely moving rat. Electroencephalogr Clin Neurophysiol 54:203–219. 10.1016/0013-4694(82)90162-6 6179745

[B62] Longair MH, Baker DA, Armstrong JD (2011) Simple neurite tracer: open source software for reconstruction, visualization and analysis of neuronal processes. Bioinformatics 27:2453–2454. 10.1093/bioinformatics/btr390 21727141

[B63] Mandyam CD, Wee S, Crawford EF, Eisch AJ, Richardson HN, Koob GF (2008) Varied access to intravenous methamphetamine self-administration differentially alters adult hippocampal neurogenesis. Biol Psychiatry 64:958–965. 10.1016/j.biopsych.2008.04.010 18490002 PMC2587157

[B64] Maurer AP, Vanrhoads SR, Sutherland GR, Lipa P, McNaughton BL (2005) Self-motion and the origin of differential spatial scaling along the septo-temporal axis of the hippocampus. Hippocampus 15:841–852. 10.1002/hipo.20114 16145692

[B65] Merker B (2013) Cortical gamma oscillations: the functional key is activation, not cognition. Neurosci Biobehav Rev 37:401–417. 10.1016/j.neubiorev.2013.01.013 23333264

[B66] Milczarek MM, Vann SD, Sengpiel F (2018) Spatial memory engram in the mouse retrosplenial cortex. Curr Biol 28:1975–1980.e6. 10.1016/j.cub.2018.05.002 29887312 PMC6013279

[B67] Mitra P, Bokil H (2008) Observed brain dynamics. Oxford: Oxford UP.

[B68] Moser MB, Trommald M, Andersen P (1994) An increase in dendritic spine density on hippocampal CA1 pyramidal cells following spatial learning in adult rats suggests the formation of new synapses. Proc Natl Acad Sci U S A 91:12673–12675. 10.1073/pnas.91.26.12673 7809099 PMC45501

[B69] Moser MB, Trommald M, Egeland T, Andersen P (1997) Spatial training in a complex environment and isolation alter the spine distribution differently in rat CA1 pyramidal cells. J Comp Neurol 380:373–381. 10.1002/(SICI)1096-9861(19970414)380:3<373::AID-CNE6>3.0.CO;2-# 9087519

[B70] Nelson AJ, Vann SD (2014) Mammillothalamic tract lesions disrupt tests of visuo-spatial memory. Behav Neurosci 128:494–503. 10.1037/bne0000001 24956013 PMC4105316

[B71] Nelson AJ, Vann SD (2017) The importance of mammillary body efferents for recency memory: towards a better understanding of diencephalic amnesia. Brain Struct Funct 222:2143–2156. 10.1007/s00429-016-1330-x 27783220 PMC5504269

[B72] Nokia MS, Sisti HM, Choksi MR, Shors TJ (2012) Learning to learn: theta oscillations predict new learning, which enhances related learning and neurogenesis. PLoS One 7:e31375. 10.1371/journal.pone.0031375 22348078 PMC3277498

[B73] Olvera-Cortés E, Cervantes M, González-Burgos I (2002) Place-learning, but not cue-learning training, modifies the hippocampal theta rhythm in rats. Brain Res Bull 58:261–270. 10.1016/S0361-9230(02)00769-4 12128151

[B74] Orr G, Rao G, Houston FP, McNaughton BL, Barnes CA (2001) Hippocampal synaptic plasticity is modulated by theta rhythm in the fascia dentata of adult and aged freely behaving rats. Hippocampus 11:647–654. 10.1002/hipo.1079 11811658

[B75] Osher S, Shen J (2000) Digitized PDE method for data restoration. In: Analytic-computational methods in applied mathematics (Anastassiou G, ed), pp 751–771. New York: Chapman Hall.

[B76] Özyurt J, Thiel CM, Lorenzen A, Gebhardt U, Calaminus G, Warmuth-Metz M, Müller HL (2014) Neuropsychological outcome in patients with childhood craniopharyngioma and hypothalamic involvement. J Pediatr 164:876–881.e4. 10.1016/j.jpeds.2013.12.010 24507865

[B77] Pan WX, McNaughton N (1997) The medial supramammillary nucleus, spatial learning and the frequency of hippocampal theta activity. Brain Res 764:101–108. 10.1016/S0006-8993(97)00431-9 9295198

[B78] Papez JW (1937) A proposed mechanism of emotion. Arch Neurol Psychiatry 38:725–743. 10.1001/archneurpsyc.1937.02260220069003 7711480

[B79] Paxinos G, Watson C (1998) The rat brain in stereotaxic coordinates. San Diego: Academic. 10.1016/0165-0270(80)90021-7 6110810

[B80] Perry JC, Pakkenberg B, Vann SD (2019) Striking reduction in neurons and glial cells in anterior thalamic nuclei of older patients with Down syndrome. Neurobiol Aging 75:54–61. 10.1016/j.neurobiolaging.2018.11.009 30550978 PMC6357872

[B81] Pierpaoli C, Basser PJ (1996) Toward a quantitative assessment of diffusion anisotropy. Magn Reson Med 36:893–906. 10.1002/mrm.1910360612 8946355

[B82] Ray S, Maunsell JH (2011) Different origins of gamma rhythm and high-gamma activity in macaque visual cortex. PLoS Biol 9:e1000610. 10.1371/journal.pbio.1000610 21532743 PMC3075230

[B83] Renouard L, Billwiller F, Ogawa K, Clément O, Camargo N, Abdelkarim M, Gay N, Scoté-Blachon C, Touré R, Libourel PA, Ravassard P, Salvert D, Peyron C, Claustrat B, Léger L, Salin P, Malleret G, Fort P, Luppi PH (2015) The supramammillary nucleus and the claustrum activate the cortex during REM sleep. Sci Adv 1:e1400177. 10.1126/sciadv.1400177 26601158 PMC4640625

[B84] Richard GR, Titiz A, Tyler A, Holmes GL, Scott RC, Lenck-Santini PP (2013) Speed modulation of hippocampal theta frequency correlates with spatial memory performance. Hippocampus 23:1269–1279. 10.1002/hipo.22164 23832676 PMC5410367

[B85] Rogerson T, Cai DJ, Frank A, Sano Y, Shobe J, Lopez-Aranda MF, Silva AJ (2014) Synaptic tagging during memory allocation. Nat Rev Neurosci 15:157–169. 10.1038/nrn3667 24496410 PMC3992944

[B86] Sagi Y, Tavor I, Hofstetter S, Tzur-Moryosef S, Blumenfeld-Katzir T, Assaf Y (2012) Learning in the fast lane: new insights into neuroplasticity. Neuron 73:1195–1203. 10.1016/j.neuron.2012.01.025 22445346

[B87] Sarra S (2006) Digital total variation filtering as postprocessing for Chebyshev pseudospectral methods for conservation laws. Numerical Algorithms 41:17–33. 10.1007/s11075-005-9003-5

[B88] Savastano LE, Hollon TC, Barkan AL, Sullivan SE (2018) Korsakoff syndrome from retrochiasmatic suprasellar lesions: rapid reversal after relief of cerebral compression in 4 cases. J Neurosurg 128:1731–1736. 10.3171/2017.1.JNS162719 28574307

[B89] Savage LM, Hall JM, Vetreno RP (2011) Anterior thalamic lesions alter both hippocampal-dependent behavior and hippocampal acetylcholine release in the rat. Learn Mem 18:751–758. 10.1101/lm.023887.111 22086393 PMC3222893

[B90] Scheffer-Teixeira R, Tort AB (2017) Unveiling fast field oscillations through comodulation. eNeuro 4:ENEURO.0079–17.2017. 10.1523/ENEURO.0079-17.2017 28785730 PMC5545523

[B91] Scheffer-Teixeira R, Belchior H, Leão RN, Ribeiro S, Tort AB (2013) On high-frequency field oscillations (>100 Hz) and the spectral leakage of spiking activity. J Neurosci 33:1535–1539. 10.1523/JNEUROSCI.4217-12.2013 23345227 PMC6618720

[B92] Schindelin J, Arganda-Carreras I, Frise E, Kaynig V, Longair M, Pietzsch T, Preibisch S, Rueden C, Saalfeld S, Schmid B, Tinevez JY, White DJ, Hartenstein V, Eliceiri K, Tomancak P, Cardona A (2012) Fiji: an open-source platform for biological-image analysis. Nat Methods 9:676–682. 10.1038/nmeth.2019 22743772 PMC3855844

[B93] Sharp PE, Koester K (2008) Lesions of the mammillary body region alter hippocampal movement signals and theta frequency: implications for path integration models. Hippocampus 18:862–878. 10.1002/hipo.20474 18702112

[B94] Sheremet A, Burke SN, Maurer AP (2016) Movement enhances the nonlinearity of hippocampal theta. J Neurosci 36:4218–4230. 10.1523/JNEUROSCI.3564-15.2016 27076421 PMC4829647

[B95] Sheremet A, Kennedy JP, Qin Y, Zhou Y, Lovett SD, Burke SN, Maurer AP (2019) Theta-gamma cascades and running speed. J Neurophysiol 121:444–458. 10.1152/jn.00636.2018 30517044 PMC6397401

[B96] Slawinska U, Kasicki S (1998) The frequency of rat's hippocampal theta rhythm is related to the speed of locomotion. Brain Res 796:327–331. 10.1016/S0006-8993(98)00390-4 9689489

[B97] Sorzano CO, Thévenaz P, Unser M (2005) Elastic registration of biological images using vector-spline regularization. IEEE Trans Biomed Eng 52:652–663. 10.1109/TBME.2005.844030 15825867

[B98] Talk A, Kang E, Gabriel M (2004) Independent generation of theta rhythm in the hippocampus and posterior cingulate cortex. Brain Res 1015:15–24. 10.1016/j.brainres.2004.04.051 15223362

[B99] Tavor I, Hofstetter S, Assaf Y (2013) Micro-structural assessment of short term plasticity dynamics. Neuroimage 81:1–7. 10.1016/j.neuroimage.2013.05.050 23702416

[B100] Tort AB, Komorowski R, Eichenbaum H, Kopell N (2010) Measuring phase-amplitude coupling between neuronal oscillations of different frequencies. J Neurophysiol 104:1195–1210. 10.1152/jn.00106.2010 20463205 PMC2941206

[B101] Tronel S, Fabre A, Charrier V, Oliet SH, Gage FH, Abrous DN (2010) Spatial learning sculpts the dendritic arbor of adult-born hippocampal neurons. Proc Natl Acad Sci U S A 107:7963–7968. 10.1073/pnas.0914613107 20375283 PMC2867872

[B102] Tsanov M, Manahan-Vaughan D (2009) Long-term plasticity is proportional to theta-activity. PLoS One 4:e5850. 10.1371/journal.pone.0005850 19513114 PMC2688745

[B103] Tsanov M, Wright N, Vann SD, Erichsen JT, Aggleton JP, O'Mara SM (2011) Hippocampal inputs mediate theta-related plasticity in anterior thalamus. Neuroscience 187:52–62. 10.1016/j.neuroscience.2011.03.055 21459129 PMC3855191

[B104] Van der Werf YD, Witter MP, Uylings HB, Jolles J (2000) Neuropsychology of infarctions in the thalamus: a review. Neuropsychologia 38:613–627. 10.1016/S0028-3932(99)00104-9 10689038

[B105] Van der Werf YD, Scheltens P, Lindeboom J, Witter MP, Uylings HB, Jolles J (2003) Deficits of memory, executive functioning and attention following infarction in the thalamus: a study of 22 cases with localised lesions. Neuropsychologia 41:1330–1344. 10.1016/S0028-3932(03)00059-9 12757906

[B106] Vann SD (2009) Gudden's ventral tegmental nucleus is vital for memory: re-evaluating diencephalic inputs for amnesia. Brain 132:2372–2384. 10.1093/brain/awp175 19602577

[B107] Vann SD (2010) Re-evaluating the role of the mammillary bodies in memory. Neuropsychologia 48:2316–2327. 10.1016/j.neuropsychologia.2009.10.019 19879886

[B108] Vann SD (2013) Dismantling the Papez circuit for memory in rats. Elife 2:e00736. 10.7554/eLife.00736 23805381 PMC3691571

[B109] Vann SD, Aggleton JP (2003) Evidence of a spatial encoding deficit in rats with lesions of the mammillary bodies or mammillothalamic tract. J Neurosci 23:3506–3514. 10.1523/JNEUROSCI.23-08-03506.2003 12716960 PMC6742300

[B110] Vann SD, Albasser MM (2009) Hippocampal, retrosplenial, and prefrontal hypoactivity in a model of diencephalic amnesia: evidence towards an interdependent subcortical-cortical memory network. Hippocampus 19:1090–1102. 10.1002/hipo.20574 19280662

[B111] Vann SD, Nelson AJ (2015) The mammillary bodies and memory: more than a hippocampal relay. Prog Brain Res 219:163–185. 10.1016/bs.pbr.2015.03.006 26072239 PMC4498492

[B112] Vann SD, Tsivilis D, Denby CE, Quamme JR, Yonelinas AP, Aggleton JP, Montaldi D, Mayes AR (2009) Impaired recollection but spared familiarity in patients with extended hippocampal system damage revealed by 3 convergent methods. Proc Natl Acad Sci U S A 106:5442–5447. 10.1073/pnas.0812097106 19289844 PMC2664061

[B113] Vukovic J, Borlikova GG, Ruitenberg MJ, Robinson GJ, Sullivan RK, Walker TL, Bartlett PF (2013) Immature doublecortin-positive hippocampal neurons are important for learning but not for remembering. J Neurosci 33:6603–6613. 10.1523/JNEUROSCI.3064-12.2013 23575857 PMC6619068

[B114] West MJ, Slomianka L, Gundersen HJ (1991) Unbiased stereological estimation of the total number of neurons in the subdivisions of the rat hippocampus using the optical fractionator. Anat Rec 231:482–497. 10.1002/ar.1092310411 1793176

[B115] Wickham H (2016) ggplot2: Elegant Graphics for Data Analysis. New York:Springer-Verlag.

[B116] Wolff M, Vann SD (2019) The cognitive thalamus as a gateway to mental representations. J Neurosci 39:3–14. 10.1523/JNEUROSCI.0479-18.2018 30389839 PMC6325267

[B117] Yoneoka Y, Takeda N, Inoue A, Ibuchi Y, Kumagai T, Sugai T, Takeda K, Ueda K (2004) Acute Korsakoff syndrome following mammillothalamic tract infarction. AJNR Am J Neuroradiol 25:964–968. 15205131 PMC7975643

[B118] Zakowski W, Zawistowski P, Braszka L, Jurkowlaniec E (2017) The effect of pharmacological inactivation of the mammillary body and anterior thalamic nuclei on hippocampal theta rhythm in urethane-anesthetized rats. Neuroscience 362:196–205. 10.1016/j.neuroscience.2017.08.043 28844761

